# Study of patients with acute undifferentiated fever identifies dengue as a growing threat to public health in Mali

**DOI:** 10.1371/journal.pntd.0014494

**Published:** 2026-07-08

**Authors:** Lassina Doumbia, Laurence Thirion, Raphaelle Klitting, Geraldine Piorkowski, Rayane Amaral, Laura Pezzi, Yacouba Kone, Oumar Kokena, Gilda Grard, Xavier de Lamballerie, Ousmane Koita, Audrey Dubot-Pérès

**Affiliations:** 1 Laboratory for Applied Molecular Biology (LBMA), Campus de Badalabougou, University of Sciences, Techniques and Technologies of Bamako (USTTB), Bamako, Mali; 2 Unité des Virus Émergents (UVE: Aix-Marseille Univ, Università di Corsica, Inserm, IRBA), Marseille, France; 3 Centre National de Référence des Arbovirus, Inserm-IRBA, Marseille, France; 4 Clinique Espoir, Bamako, Mali; Monash University, AUSTRALIA

## Abstract

The circulation of arboviruses in sub-Saharan African countries remains poorly documented. The associated health burden may be underestimated and masked by the significance of malaria. Here, we have investigated acute undifferentiated fevers for arboviral infections in Mali (2016–2024). To estimate the proportion of patients with arboviral infection, and in particular dengue. A retrospective (2016–2022) and a prospective (2023–2024) studies were conducted in patients from health centers and hospitals of Mali (mainly in the Bamako region) selected by health professionals. Studies included patients with acute fever lasting less than 7 days; the prospective sub-study excluding pyogenic, urinary, tuberculosis, viral hepatitis, typhoid fever and post-traumatic infections. Blood samples were tested for arboviruses using molecular detection (including serotyping) and genomic sequencing. We collected demographic data and results of malaria testing for all patients and, in the prospective study, a set of clinical data. A total of 2,022 patients were included. Dengue virus (DENV) was the most frequently detected pathogen (retrospective study: 7.6%, 16/210 patients; prospective study: 29.5%, 535/1812 patients). We also detected chikungunya virus (n = 7), West Nile virus (n = 2) and Rift Valley fever virus (n = 1). Three serotypes of dengue were identified: DENV-2 (n = 185), DENV-1 (n = 113) and DENV-3 (n = 105); 148 DENV cases could not be typed. For each serotype, phylogenetic analyses identified a major lineage recently originating from the subregion (DENV-1-III; DENV-2-II; DENV-3-III). In contrast to malaria, the dengue detection rate was higher among patients over 18 years of age. The most frequently observed symptoms were headache, asthenia, arthralgia, myalgia and back pain. The mean number of those symptoms per patient was significantly higher in dengue patients. We recorded 6 cases of hemorrhagic dengue, but no deaths and no case requiring transfer to intensive care. Our findings confirm the threat posed by arbovirus infections in Mali, and more specifically the growing burden of dengue fever on public health. Monitoring dengue fever has become a major challenge in sub-Saharan countries in order to determine the conditions necessary for the future implementation of a dengue vaccination policy tailored to the public health objectives of these countries.

## Author summary

Arboviruses are viruses spread by blood-feeding insects such as mosquitoes and ticks. Examples include dengue, chikungunya, and West Nile viruses, which can cause mild to severe fever-related illnesses. Their presence in sub-Saharan Africa is not well documented, and their impact may be underestimated because symptoms can resemble those of malaria. In this study we looked for arboviruses in blood samples from sick patients from 2016-2024 attending health centers and hospitals in the Bamako region. We analyzed samples from 2022 patients. Among the 210 samples collected between 2016–2022, 8% were positive for dengue. Among the 1812 samples collected between 2023–2024, 30% were positive for dengue. We also identified, seven patients infected with chikungunya virus, 2 with West-Nile virus and one with Rift Valley fever virus. Genetic analysis of the dengue virus showed that the infections were caused by virus strains originating from neighboring countries where similar viruses had circulated in recent years. These findings are important for public health because they reveal a recent rise in dengue virus circulation in Mali and other West African countries. Tracking arboviruses, and especially dengue, has become an important challenge for sub-Saharan African countries. It helps health authorities understand the situation and plan how a future dengue vaccination program could best protect their communities.

## Introduction

The circulation of a variety of human arboviruses has been documented for decades in sub-Saharan Africa [1,2], *e.g.,* dengue virus (DENV), Yellow fever virus (YFV), Zika virus (ZIKV), chikungunya virus (CHIKV), O’nyong-nyong virus (ONNV), Sindbis virus (SINV), Rift Valley fever virus (RVFV) or Crimean-Congo hemorrhagic fever virus (CCHFV).

Our understanding of the epidemiological situation for these viruses remains largely incomplete because: (1) a large proportion of human arboviral infections are clinically mild and non-specific, with common symptoms including fever, headache, rash, myalgia or arthralgia [3]; (2) the lack of availability of diagnostic tests in many African countries; and (3) few sero-epidemiological studies have been conducted recently, limiting our ability to assess the circulation of pathogens, their geographical distribution, and their temporal dynamics.

According to a report from the World Health Organization (WHO) [4–8], many West African countries reported confirmed cases of arbovirus infections in 2023 and 2024. For CHIKV, cases were reported in Burkina Faso (311), Mali (1), and Senegal (230); for RVFV, one case each was reported in Mauritania, Niger, and Senegal; for ZIKV, two cases in Senegal; and for YFV, two cases in Guinea and Senegal [5,7,8]. Regarding DENV, cases were reported in Burkina Faso (68,346 cases, 688 deaths), Côte d’Ivoire (321 cases, 27 deaths), Ghana (9 cases), Mali (62 cases, 1 death), Senegal (248 cases), and Togo (2 cases, 1 death). In the same year (2023), the WHO African Region recorded a burden of 782 deaths due to DENV infection across 15 countries, including Mali [4].

The overall picture confirms active arbovirus circulation. In regions where malaria remains highly prevalent, determining the actual proportion of arboviral infections, particularly dengue, among non-specific febrile illnesses remains challenging. Dengue appears to be increasing in many countries, but it is still difficult to discern whether this reflects a true rise in incidence or improved diagnostic capacity.

Here, we report results from 2,022 patients presenting with fever in health centers of Mali. Advanced molecular diagnostic tests were performed for the most common arboviruses diseases, in addition to malaria testing.

A major strength of this study lies in its ability to compare the frequency of dengue cases between two periods (April 2016 to October 2022 and November 2022 to December 2024), and, for the most recent period, to analyze medical and eco-demographic data collected through a dedicated questionnaire in a region where baseline epidemiological data on dengue were previously scare.

## Results

### Retrospective sub-study (2016–2022)

#### Patient characteristics.

Among the 210 included patients, age ranged from 2 to 69 years (median age: 19 years old (yo); IQR: 9–29); the male/female ratio was unbalanced in favour of women (76/134 = 0.57; *p* < .001) (Table 1).

**Table 1:** Clinical and demographic characteristics of febrile patients enrolled from April 2016 to October 2022 According to Pathogen Identification.

Characteristics, n (%)	All N = 210	DENV N = 16	CHIKV N = 4	RVFV N = 1	WNV N = 2
**Demographics**					
**Age (years)**					
Median (IQR),	19 (9 – 29)	17 (9 – 26)	**11 (6 – 17)**	NA	16 (15 – 18)
min - max	2 - 69	5 - 47	4 - 21	NA	13 - 19
**Age Groups (years)**					
< 10	58 (27.6)	5 (31.3)	2 (50.0)	1 (100)	0
10 to < 18	34 (16.2)	3 (18.8)	1 (25.0)	0	1 (50.0)
18 to < 30	68 (32.4)	6 (37.5)	1 (1.5)	0	1 (50.0)
30 to < 45	42 (20.0)	1 (6.3)	0	0	0
45 to < 60	6 (2.9)	1 (6.3)	0	0	0
> 60	2 (1.0)	0	0	0	0
**Sex**					
Male	76 (36.2)	8 (50.0)	2 (50.0)	0	2 (100)
Female	134 (63.8)	8 (50.0)	2 (50.0)	1 (100)	0
**City of Residence**					
Bamako	116 (55.2)	15 (93.8)	4 (100)	1 (100)	2 (100)
Selingue	94 (44.8)	1 (6.2)	0	0	0
Malaria Positive	13 (6.2)	3 (18.8)	0	0	0
**Year of Sample Collection**					
2016	60 (28.6)	1 (6.3)	0	0	0
2017	34 (16.2)	0	0	0	0
2019	73 (34.8)	11 (68.8)	3 (75.0)	1 (100)	2 (100)
2020	16 (7.6)	1 (6.3)	1 (25.0)	0	0
2021	15 (2.1)	1 (6.3)	0	0	0
2022	12 (5.7)	2 (12.5)	0	0	0

Except for age, all characteristics are presented in the table as the number of patients (n) with the given characteristic, with the corresponding percentage (in parentheses) calculated over the total number of patients in each group (N = 210 for all included patients, N = 16 for patients found positive by DENV RT-PCR…).

Clinical data were available for 18 patients only: headache was reported in 10/18 (55.6%) patients, myalgia in 13/18 (72.2%), arthralgia in 5/18 (27.8%), asthenia in 5/18 (27.8%), ocular symptoms in 4/18 (22.2%), hemorrhage in 3/18 (16.7%), anorexia in 2/18 (11.1%) and back pain in 1/18 (5.6%).

### Identification of pathogens

All samples tested negative for LASV, EBOV, CCHFV and MARV by RT-qPCR (Fig A in S1 Appendix). Thirteen samples (6.2%) were positive for *Plasmodium* spp.

Twenty-three samples (11%) were positive by RT-qPCR for at least one of the viruses investigated (Table 1).

DENV was detected in 16 samples (7.6%), more frequently in Bamako than in Selingue (*p* = .001, Chi^2^ test). We identified 3 serotypes (Table I in S1 Appendix). Co-infection with *Plasmodium* spp was found in 3/16 (18.8%) dengue patients (Table 1). The age distribution of DENV cases was similar to that of all tested cases. Two of the three patients with hemorrhagic history were infected by DENV. The main symptoms reported for dengue patients were myalgia in 5 patients, and headache in 4. Ocular symptom and asthenia were each reported in two dengue patients, and arthralgia in one.

Four samples (1.9%) were positive for CHIKV, 2 (1.0%) for WNV, and 1 (0.5%) for RVFV. ONNV and ZIKV were not detected and testing for YFV is presented in the discussion section. The proportion of positive samples was highest in 2019 (17/23, 73.9%), including a majority of dengue cases (11/17, 64.7%).

### Prospective Sub-study (2023–2024)

#### Patient characteristics.

Among the 1,812 included patients, age ranged from 1 to 88 yo (median age: 24 yo; IQR: 14–35) (Table 2). The male/female ratio was balanced (872/940 = 0.93) (Table 2).

**Table 2:** Clinical and demographic characteristics of febrile patients enrolled from February 2023 to December 2024 according to pathogen identification.

Characteristics, n (%)	All N = 1812	DENV N = 535	CHIKV N = 3
**Demographics**			
**Age (years)**			
Median (IQR)	24 (14 - 35)	27 (20 – 39)	35 (21 – 37)
min - max	1 - 88	1 - 87	6 - 38
**Age Groups (years)**			
< 10	330 (18.2)	41 (7.7)	1 (33.3)
10 to < 18	259 (14.3)	58 (10.8)	0
18 to < 30	558 (30.8)	194 (36.3)	0
30 to < 45	410 (22.6)	154 (28.8)	2 (66.7)
45 to < 60	167 (9.2)	56 (10.5)	0
> 60	88 (4.9)	32 (6.0)	0
**Sex**			
Male	872 (48.1)	262 (49.0)	2 (66.7)
Female	940 (51.9)	273 (51.0)	1 (33.3)
**City of Residence**			
Bamako	1643 (90.7)	486 (90.8)	3 (100)
Diema	1 (0.1)	0	0
Fourou	53 (2.9	0	0
Kenieba	27 (1.5)	19 (3.6)	0
Kita	3 (0.2)	1 (0.2)	0
Koulikoro	82 (4.5)	29 (5.4)	0
Sadiola	2 (0.1)	0	0
Sikasso	1 (0.1)	0	0
**Duration Symptoms (days)**			
< 5	1679 (92.7)	501 (93.6)	3 (100)
> 5	133 (7.3)	34 (6.4)	0
**Clinical Signs & Symptoms**			
Headache	1481 (81.7)	480 (89.7)	2 (66.7)
Asthenia	969 (53.5)	325 (60.7)	2 (66.7)
Myalgia	825 (45.5)	266 (49.7)	1 (33.3)
Arthralgia	702 (38.7)	306 (57.2)	1 (33.3)
Back pain	207 (11.4)	96 (17.9)	0
Gastroenteritis	175 (9.7)	45 (8.4)	0
Pharyngitis	74 (4.1)	14 (2.6)	0
Respiratory signs	64 (3.5)	8 (1.5)	0
Ocular symptoms	41 (2.3)	16 (3.0)	0
Rash	24 (1.3)	4 (0.7)	0
Anorexia	12 (0.7)	3 (0.6)	0
Encephalitis	10 (0.6)	0	0
Haemorrhage	6 (0.3)	3 (0.6)	0
Meningitis	2 (0.1)	0	0
Malaria Positive	71 (3.9)	15 (2.8)	0
**Year of Sample Collection**			
2023	877 (48.4)	215 (40.2)	2 (66.7)
2024	935 (51.6)	320 (59.8)	1 (33.3)

Except for age, all characteristics are presented in the table as the number of patients (n) with the given characteristic, with the corresponding percentage (in parentheses) calculated over the total number of patients in each group (N = 1812 for all included patients, N = 535 for patients found positive by DENV RT-PCR, N = 3 for patients found positive by CHIKV RT-PCR …).

The most frequently reported symptoms were headache (480), asthenia (325), arthralgia (306) and myalgia (266) (Table 2).

Table 3 shows the mean number of symptoms per patient, among headache, asthenia, myalgia, arthralgia and back pain, in the different subpopulations: patients with dengue (more details are provided below); patients with malaria; patients with both malaria and dengue; patients with no etiological diagnosis. Figure in supplemental (Fig B in S1 Appendix) shows the age distribution for the same subpopulations.

**Table 3:** Mean number of clinical symptoms (Headache, asthenia, myalgia, arthralgia and back pain) per patient in the different subpopulations.

Mean number of symptoms (for the 5 main symptoms^a^):						
Patient group	Mean	95%CI	Patient group	Mean	95%CI	*P* value^b^
Dengue POS, n = 535	2.75	2.6-2.9	No aetiological diagnosis, n = 1218	2.13	2.1-2.2	<.001
Malaria POS, n = 71	2.06	1.7-2.4	No aetiological diagnosis, n = 1218	2.13	2.1-2.2	.650
Dengue POS only, n = 520	2.76	2.7-2.9	Malaria POS only, n = 56	1.93	1.6-2.3	<.001
Dengue POS only, n = 520	2.76	2.7-2.9	Malaria & Dengue^c^, n = 15	2.53	1.8-3.2	.482

^a^Headache, asthenia, myalgia, arthralgia and back pain. ^b^ p value calculated using t test. POS: positive. POS only: excluding patients found positive for both dengue and malaria. ^c^ Patients found positive for both dengue and malaria.

### Identification of pathogens

All samples tested negative for LASV, EBOV, CCHFV, and MARV (Fig A in S1 Appendix). Seventy-one (3.9%) of the 1,812 included patients were positive for *Plasmodium* spp.

Five hundred thirty-eight samples (538/1,812; 29.7%) were positive by RT-qPCR for at least one of the viruses investigated (Table 2): 34.3% in 2024 (321/935) and 24.7% in 2023 (217/877).

DENV was detected in 535/1,812 patients (29.5%), significantly more frequently in those with age > 18 years (35.7% vs 16.8%; *p* < .001). The distribution of cases was similar in men and women (*p* = .7) and in Bamako *versus* the other sites investigated. We identified three serotypes (Table J in S1 Appendix). DENV-1 and DENV-3 were the most prevalent in 2023, while DENV-2 was dominant in 2024. Co-infection with *Plasmodium* spp was observed in 15/535 dengue patients (2.8%) (Table 2).

The most common symptoms were more frequent in DENV-positive than in DENV-negative patients: headache (89.7% *vs* 78.4%, *p* < .001); asthenia (60.7% *vs* 50.4%, *p* < .001): arthralgia (57.2% *vs* 31.0%, *p* < .001); myalgia (49.7% *vs* 43.8%, *p* < .021); back pain (17.9% *vs* 8.7%, *p* < .001). Patients with dengue infection had a higher mean number of these symptoms than dengue-negative patients: 2.75 (95%CI: 2.65-2.86) *versus* 2.12 (95%CI: 2.05-2.19), (Student t-test; *p* < .001). Of the six patients with a history of hemorrhagic symptoms, three were infected with DENV.

Three patients were positive for CHIKV. ZIKV, RVFV and WNV were not detected; testing for YFV and ONNV is presented in the Discussion section.

### Sequencing and phylogenetic analysis

For DENV, 95 complete genomes (≥9,700 nt) and 11 partial genomes (<9,700 nt) were obtained (Table K in S1 Appendix). For CHIKV, three complete genomes and one partial genome were obtained (Table K in S1 Appendix).

### DENV-1

Six sequences (13%) belong to the clade DENV-1-III-A and forty sequences (87%) to the clade DENV-1-III-A.2.

**Fig 1:**
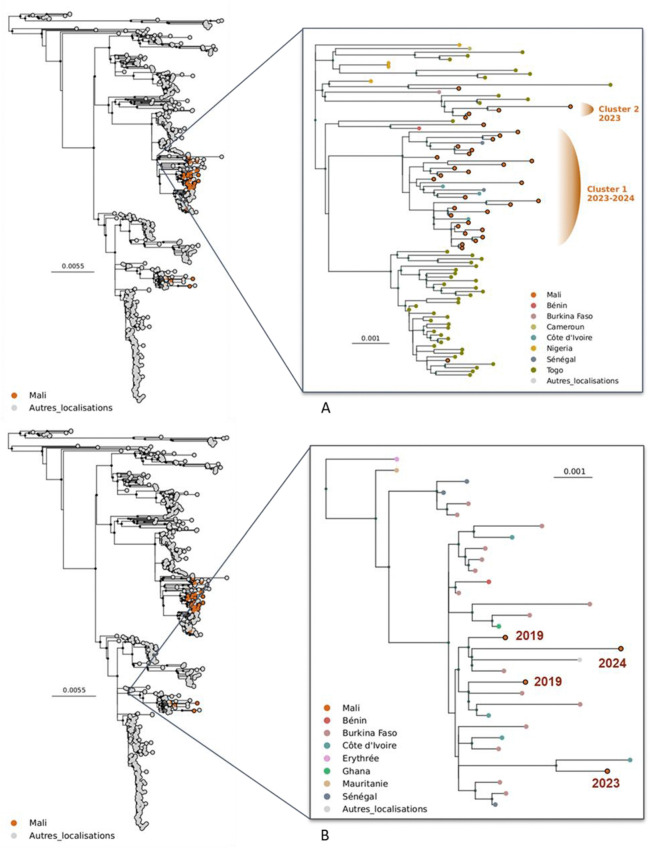
Phylogeny Analysis of DENV-1 Genotype III Sequences. Maximum likelihood (ML) phylogenies were constructed using IQ-Tree (version 1.6.12), with the best-fit model identified by ModelFinder, and branch support estimated using the ultrafast bootstrap approximation (UFBoot2, 1000 replicates). Nodes with a bootstrap support above 95 are indicated by black dots. Sequences from this study are shown in orange. Panel A shows Cluster 1 and 2, and panel B includes separate sequences from this study.

The phylogenetic analysis included 37 complete sequences from this study (Table L in S1 Appendix) (6 partial and 3 potential recombinant sequences were excluded). The majority of the sequences from the years 2023 and 2024 group in two separate clusters (Fig 1A). Cluster 1 (Bamako 2023 and 2024) encompasses most sequences (29/37), and is closely related to sequences from Côte d’Ivoire and Senegal. Cluster 2 is much smaller (4/37) and consists exclusively of sequences from Mali, rooted by sequences from Togo. The 4 remaining sequences from our study (2019, 2023 and 2024) do not group with other sequences from Mali, but with sequences from Burkina Faso, Côte d’Ivoire, and other countries (Fig 1B).

### DENV-2

One sequence (3%) belongs to the DENV-2-II-B clade; thirty-two sequences (97%) belong to the DENV-2-II-F.1.1 clade.

**Fig 2:**
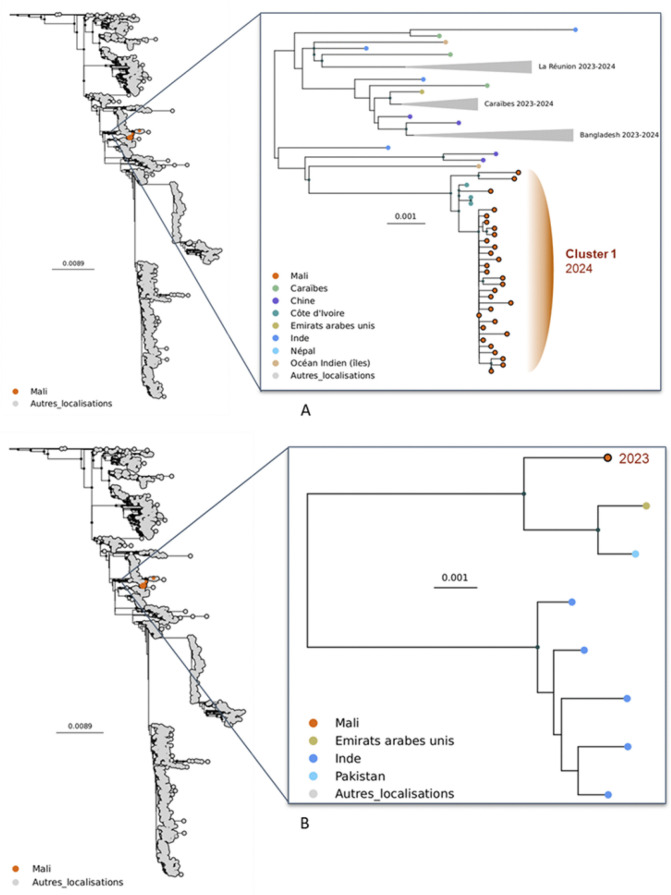
Phylogeny Analysis of DENV-2 Genotype II clade F Sequences. Maximum likelihood (ML) phylogenies were constructed using IQ-Tree (version 1.6.12), with the best-fit model identified by ModelFinder, and branch support estimated using the ultrafast bootstrap approximation (UFBoot2, 1000 replicates). Nodes with bootstrap support above 95 are shown with a black dot. Sequences from this study are shown in orange. Panel A shows a zoom on the major clusters and panel B on a single sequence that clustered separately.

We removed one partial sequence leading to a DENV-2-II-F phylogenetic analysis including 31 sequences from this study (Table M in S1 Appendix). All 2024 sequences (30) group into one major cluster related to three sequences from Côte d’Ivoire (Fig 2A), and rooted by sequences from the Indian Ocean and Asia (China, India), with an important phylogenetic distance between African and Indian Ocean/Asian sequences. The only sequence from 2023 is related to sequences from the United Arab Emirates and Pakistan (Fig 2B).

Additional phylogenetic analysis (Fig C in S1 Appendix) shows that the 2019 DENV-2-II-B sequence is closely related to sequences from Burkina Faso and Cameroon.

### DENV-3

**Fig 3:**
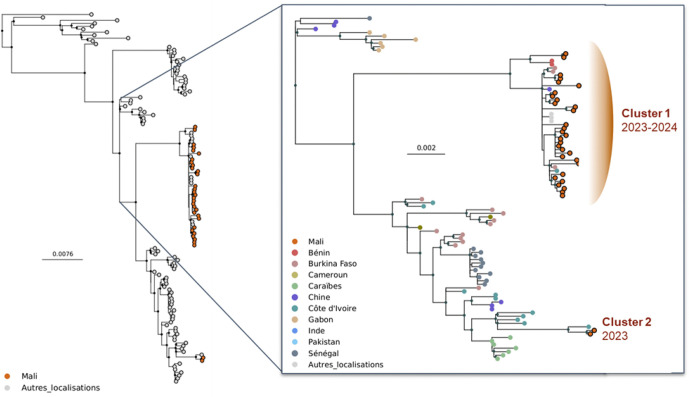
Phylogeny analysis of DENV-3 Genotype III sequences. The maximum likelihood (ML) phylogeny was constructed using IQ-Tree (version 1.6.12), with the best-fit model identified by ModelFinder, and branch support estimated using the ultrafast bootstrap approximation (UFBoot2, 1000 replicates). Nodes with a bootstrap support above 95 are shown with a black dot. Sequences from this study are shown in orange.

All 27 DENV-3 sequences from this study belong to the clade DENV-3-III-B.2. Phylogenetic analysis included 25 DENV-3 sequences from this study (Table N in S1 Appendix) (2 partial sequences were excluded). The majority of the sequences group into two clusters, cluster 1 included most sequences (23), along with sequences from other West African countries (Burkina Faso, Benin, Côte d’Ivoire, and Senegal). Cluster 2 comprises 2 sequences from our study, closely related to one from Côte d’Ivoire, and was part of a larger group encompassing sequences from Côte d’Ivoire, Burkina Faso, Senegal, Cameroun, as well as from the Caribbean (Martinique), and China (Fig 3).

### CHIKV

All four sequences from this study belong to the West African lineage. Phylogenetic analysis including three sequences (Table O in S1 Appendix) (the partial sequence was excluded) showed that two sequences are nested within a bigger clade from Côte d’Ivoire from 2023-2024, while the other fall within a clade of sequences from a recent outbreak in Senegal (Fig D in S1 Appendix).

## Discussion

To our knowledge, this study is the first in Mali to use large-scale molecular screening for arboviral pathogens. Molecular investigations were conducted in patients with AUF between April 2016 and December 2024, both for retrospective analysis of samples of interest and within a large prospective study. The retrospective sub-study revealed the presence of dengue, chikungunya and malaria cases, as well as one case of RVFV and two cases of WNV, the circulation of which had not been previously documented in Mali during the period under review. The prospective sub-study identified cases of dengue, chikungunya and malaria. Recruitment in health centers and hospitals, as carried out in our study, excluded some malaria patients treated in the first instance by general practitioners. The frequency of malaria cases among AUFs therefore cannot be assessed from our results.

The major contribution of this study is that it provides new clinical and epidemiological information on dengue fever in Mali, a subject poorly documented. Here, the most common clinical symptoms observed in dengue patients were headache, asthenia, arthralgia, myalgia, and back pain, all of which were consistently reported in previous studies in different regions of sub-Saharan Africa: Senegal [9], Gabon [10], Cameroon [11] and Niger [12]. We recorded three prospective and three retrospective cases of dengue with signs of hemorrhage, but no deaths and no manifestations requiring transfer to intensive care. However, the clinical pictures were not anecdotal, and, for example, the average number of clinical symptoms reported at the time of sampling was higher in patients with dengue than in others.

From an epidemiological perspective, dengue accounted for up to 24.5% of AUFs in 2023 and 34.2% in 2024, a situation never before reported in Mali. Additional arguments suggest a recent emergence, including: 1) the distribution of cases by age group, with a higher incidence among patients over 18 years of age, which contradicts the hypothesis of an endemic situation; 2) phylogenetic evidence indicating that the main circulating lineages emerged after 2020. Time-resolved phylogeny inference analyses suggest that the lineage at the origin of the main clusters emerged in February 2022 (95% Highest Posterior Density (HPD) interval: [2021-05-08:2022-10-12]) for DENV1 (Fig E in S1 Appendix), in November 2023 (95% HPD: 2023-05-25: 2024-04-23]) for DENV2 (Fig F in S1 Appendix), and in March 2020 (95% HPD: [2019-06-16:2021-05-26]) for DENV3 (Fig G in S1 Appendix), most often from other countries in the sub-region, suggesting that they have only recently been introduced into the West Africa. Further analysis of the minor lineages identified supports the hypothesis of multiple independent introductions. The exact origin of strains remains difficult to determine due to the low genomic sampling in Africa: prior to this study, DENV Malian sequences in GenBank included only one partial sequence and eleven sequences with genomic coverage greater than 80% [13].

This is consistent with previous reports from Africa [14] and reinforces the growing recognition of DENV circulation in West Africa. With recurrent outbreaks reported in Burkina Faso (2016–2019), Senegal (2018–2024), Mali, and neighboring countries in 2023 and 2024 [5,8,15–18], DENV has become the most frequently detected arbovirus in the region, with molecular confirmation in multiple countries including Benin, Burkina Faso, Côte d’Ivoire, Mali, Mauritania, Niger, and Senegal [12,16–22]. This aligns with WHO reports of increasing number of DENV cases across Africa, with notable outbreaks in Tanzania (6,917 cases in 2019), Senegal (248 cases in 2023) and Burkina Faso (68,346 cases in 2023) [5,8,21,23,24]. The burden of dengue in Africa may seem less severe than in other regions, particularly South Asia and the Americas. Brazil alone reported more than 10 million cases of DENV in 2024, and Bangladesh, Sri Lanka or Thailand experienced significant epidemics during the same period [25,26]. This situation could change rapidly, and estimates of the number of cases in Africa may be skewed by the recent attention given to the issue and the lack of adequate diagnostic capacity.

Importantly, we observed a pattern of simultaneous circulation of multiple strains, lineages and serotypes, reminiscent of recent reports in sub-Saharan countries: DENV-2/3/4 in Burkina Faso in 2013–2014 [20]; DENV-1/2/3 in Burkina Faso in 2017 [16]; DENV-1/2 in Côte-d’Ivoire in 2019 [21]; DENV-1/3 in Niger in 2022–2023 [12,22].

The low number of chikungunya cases does not allow conclusions to be drawn about clinical presentation and epidemiology. We note, however, that (1) molecular detection of CHIKV had never been documented previously in the literature for Mali, (2) a previous seroepidemiological study had shown the circulation of CHIKV in Mali -which is therefore confirmed [1], and (3) the strains identified all belong to the West African genotype, with at least two distinct introduction events. CHIKV strains identified in Mali are related to sequences from Burkina Faso, Côte-d’Ivoire, and Senegal, suggesting possible cross-border circulation. This suggests wider circulation than previously thought and should lead us to consider the possibility of more widespread dissemination, as was previously the case for the Eastern-Central-Southern African and Asian genotypes. Recent evidence of sustained CHIKV circulation in neighboring countries (Burkina Faso, Côte-d’Ivoire, and Senegal) [5,27,28] reinforces the likelihood of regional transmission. Co-circulation of DENV and CHIKV has been reported in other African countries, including Gabon (2007) [29], Tanzania (2018) [30] and Sudan (2022) [31].

With regard to malaria, this study does not allow for an estimation of its etiological role in AUFs in Mali during the period under consideration. It is interesting to note that 84 malaria cases including 15 co-infections malaria/dengue were found. In the prospective study, the mean number of symptoms in patients was higher in dengue patients than in patients without an etiological diagnosis (*p* < .001, t-test) or with malaria (*p* < .001, t-test). It was not higher in dengue-malaria co-infections. The age distribution of patient with no etiological diagnosis or malaria was found similar (median value at 23 and 24 years of age, respectively) and higher for dengue (27 years of age). Sow et al. (2016) reported cases of co-infection between malaria and several arboviruses, including CHIKV, YFV, ZIKV, DENV, and RVFV, in febrile patients in Senegal [32]. In agreement with previous studies in Africa [33,34], these findings suggests that arbovirus surveillance should not be limited to febrile patients who test negative for malaria.

WNV, and RVFV were detected sporadically in this study, confirming previously documented serological evidence for WNV [1] and RVFV (authors personal data). During this study, we encountered unconfirmed detections of YFV and ONNV cases; RT-qPCRs had Ct values above 36 and could not be confirmed in the absence of remaining biological material. These included six samples from the retrospective study and ten from the prospective study for YFV (vaccination against YFV is mandatory in Mali, a country where virus circulation has been documented) [23], and three samples from the prospective study for ONNV (for which serological evidence of circulation exists) [35]. We recommend that particular attention be paid to these two pathogens in future studies of AUFs in Mali.

There are a few limitations to this study. The retrospective study relies on the availability of stored samples, which were not collected using a pre-established questionnaire and may therefore have limited representativeness. The descriptive nature of the study and the lack of certain key information, such as mosquito bite history and the patient’s primary occupation, make comparisons with similar study groups difficult. The study design may have led to underrepresentation of rural populations and milder cases treated outside healthcare facilities. The frequency of test requests does not accurately represent the true burden of AUF cases in Mali. It is important to note that variations in the number of requests and confirmed cases over time may be attributed, first, to differences in the approaches of the two sub-studies, and second, to the occurrence of outbreaks.

Despite these limitations, the study provides over an extended period of eight years important insights into infectious causes of fever, identifying dengue as an emerging threat and providing a first significant set of genomic data for DENV and CHIKV in Mali.

The results of our study underscore the importance of strengthening arboviral surveillance and diagnostic capacity in Africa to better understand and respond to the threat of arbovirus infections and specifically to the growing threat of dengue. It crucial for Mali to obtain more epidemiological data on populations at risk of developing severe forms of the disease, such as pregnant women and sickle cell patients [36–39]. This information will be important in determining the conditions for the future rollout of a dengue vaccination policy tailored to the country’s public health objectives.

## Materials and methods

### Ethics statement

The study was authorized by the Malian General Directorate of Health and Public Hygiene (GDHPH) (Authorization #1868/MSDS/DGSHP/2022) and approved by the Ethics Committee of the National Institute of Public Health (NIPH) of Mali (Approval # 02/2024/CE-INSP). As part of the surveillance protocol classified as minimal-risk research, informed verbal consent was obtained from all participants prior to enrolment.

### Patient recruitment

The study involved patients with acute undifferentiated fever (AUF) recruited in health centers and hospitals of Mali, and included:

- a retrospective sub-study including samples stored in the LBMA (Laboratory for Applied Molecular Biology, University of Sciences, Techniques, and Technologies, Bamako) biobank. Left over sera samples, originally sent by health centers and hospitals to LBMA for arboviral investigations, had been stored by freezing. For the present study, samples from patients meeting the following inclusion criteria were selected: 1] age ≥ 2 years; 2] ≤5 days since symptoms onset; 3] fever ≥38.5°C. From April 2016 to October 2022, 210 patients who fulfilled the criteria were included from three health centers and three hospitals in Bamako as well as a health center in Selingue (Table A in S1 Appendix). A laboratory request form was used to collect patients’ information, including basic demographic information and nonstandard clinical data.

- a prospective sub-study with the following inclusion criteria: 1] age ≥ 1 year; 2] ≤7 days since symptoms onset; 3] reported fever or measured fever ≥37.5°C; 4] no evidence of hemorrhagic fever at the time of inclusion; 5] no evidence of infection by pyogenic pathogens, urinary tract infections, tuberculosis, viral hepatitis, typhoid fever, or post-traumatic infections; 6] no evidence of stroke or heart attack. From February 2023 to December 2024, patients who consulted the mining medical clinic in Kayes, six health centers and one hospital in Bamako, as well patients directly referred to LBMA, were included (Table B in S1 Appendix). A specific case report form was used to collect demographic and clinical data, after obtaining informed consent from the participants and assent from the children.

Blood samples from each participant were transported to the Laboratoire de Biologie Molecular Appliquée (LBMA) in Bamako, Mali for analyses and storage (Methods in S1 Appendix).

### Malaria diagnostic

Giemsa-stained thick blood films were prepared for all patients and examined using light microscopy for *Plasmodium* spp. parasite detection.

### Molecular testing

Nucleic acid extraction and RT-qPCR were first performed for hemorrhagic fever viruses, CCHFV, Lassa virus (LASV), Ebola virus (EBOV), and Marburg virus (MARV), then for CHIKV, ONNV, ZIKV, DENV, YFV, WNV, and RVFV (Table C in S1 Appendix). For DENV-positive samples, serotyping was performed using four serotype-specific amplification assays (Table C in S1 Appendix).

### Viral genome sequencing

Samples that tested positive for DENV or CHIKV by RT-qPCR with a Cq ≤ 30 were subject to a new nucleic acid extraction (methods in S1 Appendix).

Overlapping amplicons (1234–3292 bp) covering the whole genome sequence were generated as described in the supplementary methods (Tables D, E, F, G, and H in S1 Appendix). Amplicons from the same sample were pooled equimolarly and sequenced using the Ion Torrent Technologies as previously described [40].

### Sequence analysis

DENV sequences were submitted to the command-line version of Nextclade [41] for clade identification. CHIKV sequences were submitted to the Genome Detective tool [42] for genotype identification. All DENV sequences <8500 nucleotides (nt) and CHIKV sequences <9500 nt were excluded for phylogenetic analysis. Data sets used for analysis are described in supplementary methods.

Maximum likelihood phylogenetic trees were constructed using IQ-Tree (version 1.6.12), with the best-fit model identified by ModelFinder, and branch support estimated using the ultrafast bootstrap approximation (UFBoot2, 1000 replicates). A cluster was defined as any group of 2–50 sequences that includes at least one sequence from Mali and was supported by a strong bootstrap value (>95).

To evaluate the timing of emergence of the main clades identified for DENV-1-III, DENV-2-II.F1.1, and DENV-3-III.B.2, we reconstructed time-scaled phylogenies with BEAST (v1.10.551) [43] (supplemental methods in S1 Appendix).

### Statistical analysis

Data were analyzed using R software v4.0.3 (2020-10-10). The different proportions of the categorical variables were analyzed using the Chi^2^ test, or Fisher’s exact test where appropriate, the continuous variables were compared using t-test, with significance α equal to 5%.

## Supporting information

S1 Appendix Methods. Table A. Number of Patients Retrospectively Included in the Study for Each Collection Sites in Bamako and Selengue. Table B. Number of Patients Prospectively Recruited for Each Collection Sites in Bamako, Sikasso, Kayes, and Koulikoro. Table C. List of PCR Systems Used for Amplification Assays. Table D. Sequences of Primers Used for 4 Fragments Scheme Specific Amplification of Dengue Viruses. Table E. Alternative Combination of Primer Sequences Used for the Specific 9 Fragments Scheme Amplification of Dengue Virus Serotype 1. Table F. Alternative Combination of Primer Sequences Used for the Specific 8 Fragments Scheme Amplification of Dengue Virus Serotype 2. Table G. Alternative Combination of Primer Sequences Used for the Specific 8 Fragments Scheme Amplification of Dengue Virus Serotype 3. Table H. Sequences of Primers Used for Specific Amplification for Full Genome of Chikungunya Viruses in 8 Fragments. Table I. DENV Serotype Identification for Samples Collected Between 2016 and 2022. Table J. DENV Serotype Identification for Samples Collected Between February 2023 and December 2024. Table K. Sequencing Results for CHIKV and DENV-1–4. Table L. GenBank Accession Numbers and Basic Information of the DENV-1 Sequences from the Study. Table M. GenBank Accession Numbers and Basic Information of the DENV-2 Sequences from the Study. Table N. GenBank Accession Numbers and Basic Information of the DENV-3 Sequences from the Study. Table O. GenBank Accession Numbers and Basic Information of the CHIKV Sequences from the Study. Table P. List of DENV Sequences from French National Reference Center (NRC) for Arboviruses Used in Phylogenetic Analyses. Fig A. Flowchart of Study Participant’s Enrollment and Sample Processing. Fig B. Age Distribution of Patients Enrolled in the Prospective Study. Fig C. Phylogeny Analysis of DENV-2 Genotype II Clade B Sequences. Fig D. Phylogeny Analysis of CHIKV Sequences. Fig E. Root-to-tip Analysis of Sequences Used for Bayesian Inference for DENV-1. Fig F. Root-to-tip Analysis of Sequences Used for Bayesian Inference for DENV-2. Fig G. Root-to-tip Analysis of Sequences Used for Bayesian Inference for DENV-3.
(DOCX)
