# Supplementary material for: Study of patients with acute undifferentiated fever identifies dengue as a growing threat to public health in Mali
Source: PLoS Negl Trop Dis. 2026 Jul 8;20(7):e0014494. doi: 10.1371/journal.pntd.0014494 (PMC13367906; doi:10.1371/journal.pntd.0014494)
Supplement: S1 Appendix — Table A. Number of Patients Retrospectively Included in the Study for Each Collection Sites in Bamako and Selengue. Table B. Number of Patients Prospectively Recruited for Each Collection Sites in Bamako, Sikasso, Kayes, and Koulikoro. Table C. List of PCR Systems Used for Amplification Assays. Table D. Sequences of Primers Used for 4 Fragments Scheme Specific Amplification of Dengue Viruses. Table E. Alternative Combination of Primer Sequences Used for the Specific 9 Fragments Scheme Amplification of Dengue Virus Serotype 1. Table F. Alternative Combination of Primer Sequences Used for the Specific 8 Fragments Scheme Amplification of Dengue Virus Serotype 2. Table G. Alternative Combination of Primer Sequences Used for the Specific 8 Fragments Scheme Amplification of Dengue Virus Serotype 3. Table H. Sequences of Primers Used for Specific Amplification for Full Genome of Chikungunya Viruses in 8 Fragments. Table I. DENV Serotype Identification for Samples Collected Between 2016 and 2022. Table J. DENV Serotype Identification for Samples Collected Between February 2023 and December 2024. Table K. Sequencing Results for CHIKV and DENV-1–4. Table L. GenBank Accession Numbers and Basic Information of the DENV-1 Sequences from the Study. Table M. GenBank Accession Numbers and Basic Information of the DENV-2 Sequences from the Study. Table N. GenBank Accession Numbers and Basic Information of the DENV-3 Sequences from the Study. Table O. GenBank Accession Numbers and Basic Information of the CHIKV Sequences from the Study. Table P. List of DENV Sequences from French National Reference Center (NRC) for Arboviruses Used in Phylogenetic Analyses. Fig A. Flowchart of Study Participant’s Enrollment and Sample Processing. Fig B. Age Distribution of Patients Enrolled in the Prospective Study. Fig C. Phylogeny Analysis of DENV-2 Genotype II Clade B Sequences. Fig D. Phylogeny Analysis of CHIKV Sequences. Fig E. Root-to-tip Analysis of Sequences Used for Bayesian Inference for DE [file pntd.0014494.s001.docx]

**S1 Appendix**

**Methods**

**Specimen Collection and Storage**

Blood samples (1 to 5 mL), collected in EDTA or plain tubes were transported in triple packaging with an ice pack to the Laboratoire de Biologie Moléculaire Appliquée (LBMA) in Bamako, Mali. Upon receipt, the samples were centrifuged at 5,000 x g for 5 minutes at room temperature. The supernatant was used for investigation, and the remaining volumes were aliquoted and stored at -80°C at LBMA.

**Molecular Testing for Hemorrhagic Fever Viruses**

**Nucleic Acid Extraction -** Nucleic acids were extracted at LBMA from 140 µL of sample supernatant spiked with 10 µL of MS2 phage solution used as extraction internal control [1,2], using the QIAamp® Viral RNA Mini kit (Qiagen), according to the manufacturer’s instructions. The purified nucleic acid was eluted in 60 µL.

**Nucleic Acid Amplification Assays –** TaqMan probe-based, one-step, real-time reverse transcription PCR (RT-qPCR) assays were performed using the Superscript® III Platinum® One-Step qRT-PCR kit (ThermoFisher, Carlsbad, CA, USA), along with freeze-dried primers and probes (Lyoph-P&P), as previously described [3,4]. Amplification was carried out on the Opus 96™ Real-Time PCR system (Bio-Rad, Singapore). Targets included the MS2 phage internal control, CCHFV, Lassa virus (LASV), Ebola virus (EBOV), and Marburg virus (MARV) (see Table C).

A sample was considered positive for a specific virus when the controls met the defined requirements (MS2 control and positive control were positive, and the negative control remained negative), and the Cq value was < 40.

All negative samples for hemorrhagic fever viruses were further inactivated by the addition of four volumes of VXL lysis buffer (Qiagen), then frozen and shipped in triple packaging with an ice pack to UVE laboratories in Marseille, France, for additional molecular testing.

**Molecular Testing for Other Viruses**

**Nucleic Acid Extraction –** 200 µL of the abovementioned mix of serum or plasma and VXL buffer with 10 µL of MS2 phage solution were submitted to nucleic acids extraction using the QIAamp 96 DNA QIAcube HT Kit (Qiagen, Hilden, Germany), and the QIAcube HT System (QIAGEN, Hilden, Germany), according to the manufacturer’s instructions. The purified nucleic acid was eluted in 90 µL.

**Nucleic Acid Amplification Assays** – Using the same protocol as described above and the CFX 96™ Real-Time PCR system (Bio-Rad), amplification assays were carried out for: MS2 phage internal control, CHIKV, ONNV, ZIKV, DENV, YFV, WNV, and RVFV (Table C). For DENV-positive samples, serotyping was performed using four type specific amplification assays (Table C). RT-qPCR result interpretation was performed as above.

**DENV and CHIKV Genome Sequencing**

Samples that tested positive for DENV or CHIKV by RT-qPCR with a Cq ≤30 were subject to a new nucleic acid extraction. A volume of 200 µL of the abovementioned mix of sample and VXL buffer was extracted using the EZ1&2 Virus Mini kit v2.0 (QIAGEN, Germany) and the EZ1 and 2 Connect instruments (QIAGEN, Hilden, Germany) (UVE), according to the manufacturer’s instructions. Elution was performed in 60 µL. DENV and CHIKV whole genome amplifications were performed in four overlapping RT-PCRs using specific primers. In case of unsuccessful amplification, additional RT-PCRs were performed using 8 or 9 fragment scheme primers. For each amplification, the RT-PCR reaction volume was 25 µL containing 3 µL of RNAs and 0.5 µM for DENV (Table D, E, F, and G), or 0.4 µM for CHIKV (Table H), of each primer, using the Superscript® IV One-Step RT-PCR kit (Invitrogen-ThermoFisher Scientific, Baltics, UAB, Lithuania) following the manufacturer’s protocol. Reactions were performed on an Eppendorf Mastercycler X50s (Vietnam, co., Ltd) using PCR cycling conditions comprising of 10 min at 50°C, 2 min at 98°C, followed by 40 cycles of 10 s at 98°C, 10 s at [55°C for DENV, 56°C for CHIKV], and 2 min at 68°C. The final extension was 5 min at 68°C.The amplified products were visualized by agarose gel electrophoresis and subsequently purified using Monarch® PCR & DNA Cleanup Kit (New England Biolabs), according to manufacturer’s instructions.

**Sequence Analysis**

**DENV Sequence Datasets**

We first classified all sequences generated in this study using the command-line version of Nextclade [5] and identified five distinct clades: DENV-1-III.A, DENV-1-III.A.2, DENV-2-II.F.1.1, DENV-2-II.B, and DENV-3-III.B.2. All sequences of this study were filtered by size, removing any sequence with a length below 8500 nucleotides (nt).

We complemented these data by adding sequences produced by the French National Reference Center (NRC, Table P) for arboviruses that corresponded to the clades to which belonged the sequences from this study, and that were of length above 8500 nt.

To provide a genomic context for our analyses, we downloaded all sequences with a length above 8500 nucleotides available on GISAID for dengue virus 1 genotype III (accessed: 2025-06-10), dengue virus 2 genotype II (accessed: 2025-06-10), and dengue virus 3 genotype III (accessed: 2025-09-02). We used the command-line version of Nextclade to classify sequences to assign major and minor clades for all sequences in each dataset. We then selected sequences from the DENV-1-III (all clades), DENV-2-II (clades F, F.1.1 to F.1.6), DENV-2-II (clade B), DENV-3-III (clade B.2) to form four separate datasets. For each dataset, sequences were aligned using MAFFT (version 7.51142), trimmed to their coding regions, inspected manually, duplicate sequences were removed, and another step of size filtering was applied (removing any sequence with length below 8500 nt).

We added Mali and NRC sequences from Africa to each of the GISAID alignments for the DENV-1-III, DENV-2-II.B, DENV-2-II.F.1.1, DENV-3-III.B.2 dataset using MAFFT. We then removed potential recombinant sequences from each of the datasets using the Recombination Detection Program (RDP) version 4. We used RDP, GENCONV, and MAXCHI methods for primary screening and BOOTSCAN and SISCAN methods to check for recombination signals. We used the automask option to ensure optimal recombination detection.

Finally, four DENV datasets were prepared: 1) 580 sequences of DENV-1-III (532 from GISAID, 37 from this study, 11 from the French National Reference Center (NRC)), 2) 2428 sequences from DENV-2-II clades F and F.1.1 to F.1.6 (2397 from GISAID, 31 from this study), 3) 68 DENV-2-II.B sequences (67 from GISAID, 1 from this study), 4) 114 sequences from DENV-3.III-B.2 (71 from GISAID, 25 from this study, 18 from the NRC), the corresponding accession numbers of NRC are listed in Table I.

**CHIKV Sequence Dataset**

We retrieved a dataset including all sequences from CHIKV West African genotype publicly available on GenBank as of June 1^st^ 2025 and a set of sequences representative of the phylogenetic diversity within other genotypes as described previously [6], totaling 124 sequences. After filtering out sequences < 9500 nt, we added the three CHIKV sequences from this study, resulting in a final dataset of 127 sequences. As above, we then removed potential recombinant sequences from the dataset using RDP.

**Bayesian Inference of Time-Resolved Phylogenies**

To evaluate the timing of emergence of the main clades identified for DENV-1-III, DENV-2-II.F1.1, and DENV-3-III.B.2, we reconstructed time-scaled phylogenies with BEAST (v1.10.551). For each dataset, we selected a subset of sequences encompassing both the clade for which the most recent common ancestor (TMRCA) was to be estimated and a set of sequences to provide some genomic background for molecular-clock estimations, and performed a root-to-tip analysis to removed sequences whose sampling date was incongruent with their genetic divergence. We obtained subsets of 85, 49 and 97 sequences for DENV-1-III, DENV-2-II.F1.1, and DENV-3-III.B.2, respectively, which all exhibited sufficient association between genetic distances and sampling dates (⁠⁠S1 Fig E, F, and G in S1 Appendix) to perform Bayesian inference. We used the Shapiro-Rambaut-Drummond-2006 (SRD06) substitution model, with an uncorrelated lognormal (UCLN) clock model, and the Bayesian skygrid coalescent model. We ran three Markov chain Monte Carlo (MCMC) chains of 100 million states with the BEAGLE computational library. We used Tracer (v1.753) for inspecting the convergence and mixing, discarding the first 10 % of steps as burn-in, and ensuring that estimated sampling size (ESS) values associated with estimated parameters were all >200.

All GISAID/GenBank sequence IDs, alignment, xml, and tree files for this study are available at: <https://github.com/rklitting/DENV_CHIKV_Mali>.

**Table A. Number of Patients Retrospectively Included in the Study for Each Collection Sites in Bamako and Selengue.**

| Medical center name | Patient Number |
| --- | --- |
| Bamako : | |
| ASACO^a^ | 8 |
| Val De Grâce Medical cente^e^ | 1 |
| Espoir Medical Clinic^e^ | 79 |
| CSREF CV^b^ | 1 |
| Hospital^c^ | 26 |
| UHC^d^ Gabriel Touré | 1 |
| Selengue : | |
| CSREF Selingue^b^ | 94 |

^a^ Primary health center; ^b^ Secondary health center (Reference health center); ^c^ Tertiary health center (Hospital); ^d^ Quaternary Health Center (University Hospital Center); ^e^ Private health center.

**Table B. Number of Patients Prospectively Recruited for Each Collection Sites in Bamako, Sikasso, Kayes, and Koulikoro.**

| Sample collection sites | Region or district of residence of the patient | | | |  |
| --- | --- | --- | --- | --- | --- |
|  | **Bamako** | **Kayes** | **Koulikoro** | **Sikasso** | **Total** |
| Bamako : |  |  |  |  |  |
| Baflaba Medical Clinic^b,c^ | 378 | 0 | 4 | 0 | 382 |
| Danaya Medical center^b,c^ | 137 | 0 | 3 | 0 | 140 |
| DECLIC SANTE Medical center^b,c^ | 19 | 0 | 5 | 0 | 24 |
| Djiguiya Soba Medical center^b,c^ | 13 | 0 | 0 | 0 | 13 |
| Espoir Medical Clinic^b,c^ | 378 | 0 | 27 | 0 | 405 |
| LBMA^a^ | 637 | 4 | 35 | 53 | 729 |
| UHC^d^ Point G | 72 | 3 | 7 | 1 | 83 |
| Zone Espoir Medical Clinic^b,c^ | 9 | 0 | 1 | 0 | 10 |
| Kayes : |  |  |  |  |  |
| Medical Clinic^e^ | 0 | 26 | 0 | 0 | 26 |
| Total | 1643 | 33 | 82 | 54 | 1812 |

^a^ LBMA: diagnostic laboratory where some patients were directly referred; ^b^ Primary health center; ^c^ Private health center;  ^d^ Quaternary Health Center (University Hospital Center); ^e^ Mining medical clinic.

**Table C. List of PCR Systems Used for Amplification Assays.**

| Genus | Virus Name | Abbreviation | References on EVAg Catalog^a^ | References |
| --- | --- | --- | --- | --- |
| *Duinvirus* | Bacteriophage MS2 | MS2 | 001K-04235; 001K-04237 | [7] |
| *Orthonairovirus* | Crimean-Congo hemorrhagic fever virus | CCHFV | 001K-05698 | [8] |
| *Mammarenavirus* | Lassa virus | LASV | 001K-06031 | [9] |
| *Orthoebolavirus* | Ebolavirus | EBOV | 001K-05005 | [10,11]^b^ |
| *Orthomarburgvirus* | Marburg virus | MARV | 001K-04981 | [12] |
| *Alphavirus* | Chikungunya virus | CHIKV | 001K-06164 | [13,14]^b^ |
|  | O’nyong-nyong virus | ONNV | 001K-06164 | [14] |
| *Orthoflavivirus* | Dengue virus | DENV | 001K-05374 | [15,16]^b^ |
|  | Dengue virus 1, 2, 3, and 4 | DENV-1-4 | NA | [16] |
|  | Yellow fever virus | YFV | 001K-06009 | [17] |
|  | Zika virus | ZIKV | 001K-05694 | ,[18]^b^ In house |
|  | West Nile virus | WNV | 001K-05424 | [19,20]^b^ |
| *Phlebovirus* | Rift Valley fever virus | RVFV | 001K-05697 | [21,22]^b^ |

^a^ Available on <https://www.european-virus-archive.com/evag-portal/geographical-origin/marseille>; ^b^ both PCR systems were used together in a single PCR mix.

**Table D. Sequences of Primers Used for 4 Fragments Scheme Specific Amplification of Dengue Viruses.**

| Reference | Primer Name | Sequence (5’-3’) | Amplification Size (bp) |
| --- | --- | --- | --- |
| Dengue 1  (primer numbering according position of its 5’ base on DQ193572 sequence) | Den1_F15 | GTGGACCGACAAGAACAGTTTC |  |
|  | Den1_R2520 | AATTTRTATTGYTCTGTCCARGTRTG | 2506 |
|  | Den1_F2305 | AGGAWTAGGGRTYCTGCTGAC |  |
|  | Den1_R5391 | TCRGTRAARTGTGCTTCATCCAT | 3087 |
|  | Den1_F5132 | CTTCCAGCCATAGTYCGWGAGGC |  |
|  | Den1_R7740 | GACACTGCRTGTTTGRTTRTYTCTC | 2609 |
|  | Den1_F7444 | GGAGGGATCTCCAGGAAAATT |  |
|  | Den1_R10735 | AGAACCTGTTGATTCAACAGCACC | 3292 |
| Dengue 2  (primer numbering according position of its 5’ base on AY858036 sequence) | Den2_F1 | AGTWGTTAGTCTACGTGSACCGAC |  |
|  | Den2_R2876 | GARTTCCAAGCTCTRTTTGTGTTG | 2876 |
|  | Den2_F2707 | ATYATGCAGGCAGGAAAACGATC |  |
|  | Den2_R5428 | GAGTTGARATRTATCCTCTAGCTGCTAT | 2722 |
|  | Den2_F5228 | GAGGACTTCCRATAAGATACCAAACC |  |
|  | Den2_R8023 | GTGAYGAYTCCCCTATGTCACAC | 2796 |
|  | Den2_F7875 | CCTAACAAAAGGAGGACCAGGAC |  |
|  | Den2_R10723 | AGAACCTGTTGATTCAACAGCACC | 2849 |
| Dengue 3 (primer numbering according position of its 5’ base on AY923865 sequence) | Den3_F1 | AGTTGTTAGTCTACGTGGACCGAC |  |
|  | Den3_R2877 | ACCTCCCACACATTCCATGCTC | 2877 |
|  | Den3_F2709 | AAGGGAAAAGARCAYTAACACCAC |  |
|  | Den3_R5517 | TCTTGAATTGGAGCGTTGCTCTG | 2809 |
|  | Den3_F5396 | ATAGCGGCTAGAGGGTACATATC |  |
|  | Den3_R8037 | TCTTCCACTGTTGGGCTTGGTG | 2642 |
|  | Den3_F7857 | CAGAAGTGCGAGGRTACACAAAAG |  |
|  | Den3_R10707 | AGAACCTGTTGATTCAACAGCACC | 2851 |

Primers previously published [23].

**Table E. Alternative Combination of Primer Sequences Used for the Specific 9 Fragments Scheme Amplification of Dengue Virus Serotype 1.**

| Reference | Primer Name | Sequence (5’-3’) | Amplification Size (bp) |
| --- | --- | --- | --- |
|  | Den1_F15 | GTGGACCGACAAGAACAGTTTC |  |
| Dengue 1  (primer numbering according position of its 5’ base on DQ193572 sequence) | Den1_R1471^a^ | TCCGTAGTCDGTCARYTGTATTTC | 1457 |
|  | Den1_F1279^a^ | GTGYGCHAAGTTYAAGTGTGTGAC |  |
|  | Den1_R2520 | AATTTRTATTGYTCTGTCCARGTRTG | 1242 |
|  | Den1_F2305 | AGGAWTAGGGRTYCTGCTGAC |  |
|  | Den1_R3590^a^ | CAGCYARTGTWCCAGTCATSAGCAT | 1286 |
|  | Den1_F3401^a^ | GGRTGYTGGTAYGGYATGGAAAT |  |
|  | Den1_R4726^a^ | CCARCTYGGYTCCARYCTCTTC | 1326 |
|  | Den1_F4519^a^ | ATCWGGAGTSYTRTGGGACACAC |  |
|  | Den1_R5915^a^ | GGYTCCTTCCRATTCTYCCTCT | 1397 |
|  | Den1_F5726^a^ | TGGGACTAYGTYGTCACAACAGA |  |
|  | Den1_R7075^a^ | CCATCCYTTRTCRAGTCCCATCAA | 1350 |
|  | Den1_F6815^a^ | ACAGTRGCAGCYAATGAGATGG |  |
|  | Den1_R8246^a^ | CTGTYCCRCATGARACCCARTAC | 1432 |
|  | Den1_F8006^a^ | TGTGAYATYGGWGARTCCTCTCC |  |
|  | Den1_R9369^a^ | CCTCTCTGRTCWCGYCTGGATAT | 1364 |
|  | Den1_F9122^a^ | ATACTCAGAGRCATATCAAAGAT |  |
|  | Den1_R10735 | AGAACCTGTTGATTCAACAGCACC | 1614 |

The greyed-out primers are common to the 4-fragment scheme. ^a^ Primers designed by G. Piorkowski.

**Table F. Alternative Combination of Primer Sequences Used for the Specific 8 Fragments Scheme Amplification of Dengue Virus Serotype 2.**

| Reference | Primer Name | Sequence (5’-3’) | Amplification Size (bp) |
| --- | --- | --- | --- |
|  | Den2_F1 | AGTWGTTAGTCTACGTGSACCGAC |  |
| Dengue 2  (primer numbering according position of its 5’ base on AY858036 sequence) | Den2_R1271^a^ | CATAGCACAGGTCACAATGCC | 1290 |
|  | Den2_F1219^a^ | CTCCATGGTAGACAGAGGATG |  |
|  | Den2_R2876 | GARTTCCAAGCTCTRTTTGTGTTG | 1659 |
|  | Den2_F2707 | ATYATGCAGGCAGGAAAACGATC |  |
|  | Den2_R3972^a^ | GTGCARCTCACCTTCCATGC | 1280 |
|  | Den2_F3744^a^ | GCAGCTGGACTACTCTTGAG |  |
|  | Den2_R5428 | GAGTTGARATRTATCCTCTAGCTGCTAT | 1690 |
|  | Den2_F5228 | GAGGACTTCCRATAAGATACCAAACC |  |
|  | Den2_R6694^a^ | CAGTATTATTGAAGCTGCTATCC | 1481 |
|  | Den2_F6506^a^ | CGTACAATCATGCTCTCAGTG |  |
|  | Den2_R8023 | GTGAYGAYTCCCCTATGTCACAC | 1530 |
|  | Den2_F7875 | CCTAACAAAAGGAGGACCAGGAC |  |
|  | Den2_R9351^a^ | TCTCGATATGATGTCCATTACTG | 1480 |
|  | Den2_F9307^a^ | GTGCAGAGACCAACACCAAG |  |
|  | Den2_R10723 | AGAACCTGTTGATTCAACAGCACC | 1417 |

The greyed-out primers are common to the 4-fragment scheme. ^a^ Primers designed by G. Piorkowski.

**Table G. Alternative Combination of Primer Sequences Used for the Specific 8 Fragments Scheme Amplification of Dengue Virus Serotype 3.**

| Reference | Primer Name | Sequence (5’-3’) | Amplification Size (bp) |
| --- | --- | --- | --- |
| Dengue 3 (primer numbering according position of its 5’ base on AY923865 sequence) | Den3_F1 | AGTTGTTAGTCTACGTGGACCGAC |  |
|  | Den3_R1710^a^ | TGCATTGCTCCCTCTTGCGAT | 1710 |
|  | Den3_F1579^a^ | GCTCCTGATGTCCATGGTAGA |  |
|  | Den3_R2877 | ACCTCCCACACATTCCATGCTC | 1299 |
|  | Den3_F2709 | AAGGGAAAAGARCAYTAACACCAC |  |
|  | Den3_R4074^a^ | GGAACTCCCATAGCTGCCAC | 1366 |
|  | Den3_F4009^a^ | GTGCCAGTCTTCGAGCATGAG |  |
|  | Den3_R5517 | TCTTGAATTGGAGCGTTGCTCTG | 1509 |
|  | Den3_F5396 | ATAGCGGCTAGAGGGTACATATC |  |
|  | Den3_R6626^a^ | TGAGTCCTATTGAAGTCTTTCC | 1231 |
|  | Den3_F6525^a^ | TGGAAACACTCCTACTCTTGG |  |
|  | Den3_R8037 | TCTTCCACTGTTGGGCTTGGTG | 1513 |
|  | Den3_F7857 | CAGAAGTGCGAGGRTACACAAAAG |  |
|  | Den3_R9171^a^ | CCAGCTGTGTCATCAGCATAC | 1315 |
|  | Den3_F9041^a^ | GAAGACCACTGGTTCTCGCGTG |  |
|  | Den3_R10707 | AGAACCTGTTGATTCAACAGCACC | 1667 |

The greyed-out primers are common to the 4-fragment scheme. ^a^ Primers designed by G. Piorkowski.

**Table H. Sequences of Primers Used for Specific Amplification for Full Genome of Chikungunya Viruses in 8 Fragments.**

| Reference | Primer name | Sequence (5’-3’) | Amplification Size (bp) |
| --- | --- | --- | --- |
| Chikungunya virus (primer numbering according position of its 5’ base on DQ443544 sequence) | Chik_F1S | ATGGCTGCGTGAGACACAC |  |
|  | Chik_R1526 | TGTACGGGATCAAATCAGTC | 1526 |
|  | Chik_F1351 | GCAGAAGACACACACGGTCT |  |
|  | Chik_R3183 | TGGGACCACTGCCTGTCGTTTA | 1833 |
|  | Chik_F3098 | GACCTTTGACACGTTCCAGA |  |
|  | Chik_R4609 | CACTCGGATGATATCGCAGT | 1512 |
|  | Chik_F4575 | CAAGTGGAACTGCTAGACGA |  |
|  | Chik_R6340 | AACATTAAATACCGCTGAAT | 1766 |
|  | Chik_F6265 | ACACGCTGCAGAACGTACTGGCT |  |
|  | Chik_R7749 | AGGCTTCTGTTGAGGTACCG | 1485 |
|  | Chik_F7711 | CCAGCTGATCTCTGCAGTCA |  |
|  | Chik_R8919 | TGTGCATGTGTGGCTGATCTTTCT | 1209 |
|  | Chik_F8825 | CAGCACCGTGCACGATCACCGG |  |
|  | Chik_R10516 | TGTCAAAAGGTGTCCAGGCGG | 1692 |
|  | Chik_F10431 | GAAACAACATTACCGTAGCTGCC |  |
|  | Chik_R11308 | GCCTGCTGAACGACACGCAT | 877 |

Primers designed by G. Piorkowski.

**Table I. DENV Serotype Identification for Samples Collected Between 2016 and 2022.**

| Year of sample collection | Serotype, No. | | |  |
| --- | --- | --- | --- | --- |
|  | **DENV-1** | **DENV-2** | **DENV-3** | **UKN^a^** |
| 2016, n =1 | 0 | 0 | 0 | 1 |
| 2019, n=11 | 6 | 1 | 3 | 1 |
| 2020, n=1 | 0 | 0 | 0 | 1 |
| 2021, n=1 | 0 | 0 | 0 | 1 |
| 2022, n=2 | 0 | 1 | 0 | 1 |

^a^ UKN: Serotyping was not possible due to insufficient sample volume or low viral load.

**Table J. DENV Serotype Identification for Samples Collected Between February 2023 and December 2024.**

| Year of sample collection | Serotype, No. | | | |
| --- | --- | --- | --- | --- |
|  | **DENV-1** | **DENV-2** | **DENV-3** | **UKN^a^** |
| 2023, n=215 | 66 | 1 | 85 | 63 |
| 2024, n=320 | 41 | 182 | 17 | 80 |

^a^ UKN: Serotyping was not possible due to insufficient sample volume or low viral load.

**Table K. Sequencing Results for CHIKV and DENV-1-4.**

| **Virus, No.** | **RT-qPCR Positive** | **Submitted to Sequencing** | **Complete Sequencing** | **Partial Sequencing** | **Not Sequenced** |
| --- | --- | --- | --- | --- | --- |
| DENV-1 | 113 | 46 | 40 | 6 | 0 |
| DENV-2 | 185 | 34 | 32 | 1 | 1 |
| DENV-3 | 105 | 29 | 23 | 4 | 2 |
| CHIKV | 7 | 4 | 3 | 1 | 0 |
| Total | 410 | 113 | 98 | 12 | 3 |

**Table L. GenBank Accession Numbers and Basic Information of the DENV-1 Sequences from the Study.**

| Order | ID Isolate DENV-1 | Collection Date | Isolation Source | Genotype | Size (bp) | GenBank Accession Numbers |
| --- | --- | --- | --- | --- | --- | --- |
| 1 | MA08 | 11/7/2019 | Bamako | 1III_A | 10,645 | PX454579 |
| 2 | MA136 | 11/7/2019 | Bamako | 1III_A | 10,560 | PX454580 |
| 3 | MA137 | 11/7/2019 | Bamako | 1III_A | 10,360 | PX454581 |
| 4 | MA555 | 11/7/2023 | Bamako | 1III_A | 10,645 | PX454582 |
| 5 | MA562 | 11/8/2023 | Koulikoro | 1III_A.2 | 10,561 | PX454583 |
| 6 | MA592 | 11/10/2023 | Bamako | 1III_A.2 | 10,643 | PX454584 |
| 7 | MA594 | 11/13/2023 | Bamako | 1III_A.2 | 10,643 | PX454585 |
| 8 | MA602 | 11/13/2023 | Koulikoro | 1III_A.2 | 10,562 | PX454586 |
| 9 | MA648 | 11/15/2023 | Bamako | 1III_A.2 | 10,543 | PX454587 |
| 10 | MA655 | 11/11/2023 | Bamako | 1III_A.2 | 10,562 | PX454588 |
| 11 | MA681-0-1^a^; MA681-0-2^b^ | 11/17/2023 | Kenieba | 1III_A.2 | 1,400;  2,555 | PX463156; PX463157 |
| 12 | MA683 | 11/17/2023 | Kenieba | 1III_A.2 | 9,274 | PX454589 |
| 13 | MA693 | 11/17/2023 | Koulikoro | 1III_A.2 | 3,009 | NA^c^ |
| 14 | MA702 | 11/18/2023 | Bamako | 1III_A.2 | 10,561 | PX454590 |
| 15 | MA720 | 11/20/2023 | Bamako | 1III_A.2 | 10,640 | PX454591 |
| 16 | MA729 | 11/21/2023 | Bamako | 1III_A.2 | 10,597 | PX454593 |
| 17 | MA745 | 11/21/2023 | Bamako | 1III_A.2 | 10,640 | PX454594 |
| 18 | MA760 | 11/23/2023 | Koulikoro | 1III_A.2 | 10,640 | PX454596 |
| 19 | MA761 | 11/23/2023 | Bamako | 1III_A.2 | 10,641 | PX454597 |
| 20 | MA767 | 11/24/2023 | Bamako | 1III_A.2 | 10,615 | PX454598 |
| 21 | MA778 | 11/25/2023 | Koulikoro | 1III_A.2 | 10,641 | PX454599 |
| 22 | MA787 | 11/25/2023 | Bamako | 1III_A.2 | 10,562 | PX454600 |
| 23 | MA792 | 11/26/2023 | Bamako | 1III_A.2 | 10,617 | PX454601 |
| 24 | MA802 | 11/27/2023 | Bamako | 1III_A.2 | 10,644 | PX454602 |
| 25 | MA815 | 11/29/2023 | Bamako | 1III_A.2 | 10,617 | PX454603 |
| 26 | MA831 | 11/30/2023 | Bamako | 1III_A.2 | 10,642 | PX454604 |
| 27 | MA881 | 12/4/2023 | Bamako | 1III_A.2 | 10,643 | PX454605 |
| 28 | MA899 | 12/6/2023 | Bamako | 1III_A.2 | 10,616 | PX454606 |
| 29 | MA1028 | 12/19/2023 | Bamako | 1III_A.2 | 10,641 | PX454608 |
| 30 | MA1400 | 9/1/2023 | Bamako | 1III_A | 10,565 | PX454609 |
| 31 | MA1451 | 10/10/2024 | Bamako | 1III_A.2 | 10,641 | PX454611 |
| 32 | MA1452 | 10/17/2024 | Bamako | 1III_A.2 | 10,644 | PX454612 |
| 33 | MA1454 | 10/30/2024 | Bamako | 1III_A.2 | 10,643 | PX454613 |
| 34 | MA1460 | 11/9/2024 | Bamako | 1III_A.2 | 10,691 | PX454614 |
| 35 | MA1462 | 11/13/2024 | Bamako | 1III_A.2 | 10,644 | PX454615 |
| 36 | MA1466 | 11/16/2024 | Bamako | 1III_A.2 | 10,641 | PX454616 |
| 37 | MA1469 | 11/20/2024 | Bamako | 1III_A.2 | 10,662 | PX454617 |
| 38 | MA1470 | 11/21/2024 | Bamako | 1III_A.2 | 10,640 | PX454618 |
| 39 | MA1472 | 11/26/2024 | Bamako | 1III_A.2 | 10,642 | PX454619 |
| 40 | MA1473 | 11/27/2024 | Bamako | 1III_A.2 | 10,643 | PX454620 |
| 41 | MA1475 | 12/4/2024 | Bamako | 1III_A.2 | 10,561 | PX454621 |
| 42 | MA1478 | 12/12/2024 | Bamako | 1III_A.2 | 10,640 | PX454622 |

^a^ and ^b^ Two different partial sequences from the same isolate; ^c^ partial sequences with several gaps so could not be submitted to GenBank.

**Table M. GenBank Accession Numbers and Basic Information of the DENV-2 Sequences from the Study.**

| Order | ID Isolate DENV-2 | Collection Date | Isolation Source | Genotype | Size (bp) | GenBank Accession Numbers |
| --- | --- | --- | --- | --- | --- | --- |
| 1 | MA16 | 11/11/2019 | Bamako | 2II_B | 10,661 | PX454623 |
| 2 | MA663 | 11/16/2023 | Bamako | 2II_F.1.1 | 10,550 | PX454624 |
| 3 | MA1468 | 11/19/2024 | Bamako | 2II_F.1.1 | 10,666 | PX454625 |
| 4 | MA1480 | 9/27/2024 | Bamako | 2II_F.1.1 | 10,666 | PX454626 |
| 5 | MA1485 | 10/24/2024 | Bamako | 2II_F.1.1 | 10,666 | PX454627 |
| 6 | MA1486 | 10/26/2024 | Bamako | 2II_F.1.1 | 10,666 | PX454628 |
| 7 | MA1487 | 10/27/2024 | Bamako | 2II_F.1.1 | 10,666 | PX454629 |
| 8 | MA1490 | 10/30/2024 | Bamako | 2II_F.1.1 | 10,666 | PX454630 |
| 9 | MA1492 | 11/5/2024 | Bamako | 2II_F.1.1 | 10,666 | PX454631 |
| 10 | MA1498 | 11/7/2024 | Bamako | 2II_F.1.1 | 10,666 | PX454632 |
| 11 | MA1500 | 11/7/2024 | Bamako | 2II_F.1.1 | 10,666 | PX454633 |
| 12 | MA1502 | 11/7/2024 | Bamako | 2II_F.1.1 | 10,666 | PX454634 |
| 13 | MA1504 | 11/8/2024 | Bamako | 2II_F.1.1 | 10,666 | PX454635 |
| 14 | MA1511 | 11/10/2024 | Bamako | 2II_F.1.1 | 10,666 | PX454636 |
| 15 | MA1526 | 11/14/2024 | Bamako | 2II_F.1.1 | 10,666 | PX454637 |
| 16 | MA1532 | 11/15/2024 | Bamako | 2II_F.1.1 | 10,656 | PX454638 |
| 17 | MA1535 | 11/15/2024 | Bamako | 2II_F.1.1 | 10,666 | PX454639 |
| 18 | MA1541 | 11/17/2024 | Bamako | 2II_F.1.1 | 10,666 | PX454640 |
| 19 | MA1546 | 11/16/2024 | Bamako | 2II_F.1.1 | 10,663 | PX454641 |
| 20 | MA1549 | 11/18/2024 | Bamako | 2II_F.1.1 | 10,666 | PX454642 |
| 21 | MA1550 | 11/19/2024 | Bamako | 2II_F.1.1 | 10,666 | PX454643 |
| 22 | MA1551 | 11/19/2024 | Bamako | 2II_F.1.1 | 10,664 | PX454644 |
| 23 | MA1559 | 11/20/2024 | Bamako | 2II_F.1.1 | 10,666 | PX454645 |
| 24 | MA1561 | 11/20/2024 | Bamako | 2II_F.1.1 | 10,666 | PX454646 |
| 25 | MA1563 | 11/21/2024 | Bamako | 2II_F.1.1 | 10,666 | PX454647 |
| 26 | MA1567 | 11/21/2024 | Bamako | 2II_F.1.1 | 10,665 | PX454648 |
| 27 | MA1568 | 11/22/2024 | Bamako | 2II_F.1.1 | 10,666 | PX454649 |
| 28 | MA1572 | 11/22/2024 | Bamako | 2II_F.1.1 | 10,579 | PX454650 |
| 29 | MA1580 | 11/25/2024 | Bamako | 2II_F.1.1 | 10,666 | PX454651 |
| 30 | MA1582 | 11/25/2024 | Bamako | 2II_F.1.1 | 10,666 | PX454652 |
| 31 | MA1593 | 11/28/2024 | Bamako | 2II_F.1.1 | 9,446 | PX454653 |
| 32 | MA1601 | 12/1/2024 | Bamako | 2II_F.1.1 | 10,666 | PX454654 |
| 33 | MA1611 | 12/10/2024 | Bamako | 2II_F.1.1 | 10,666 | PX454655 |

**Table N. GenBank Accession Numbers and Basic Information of the DENV-3 Sequences from the Study.**

| Order | ID Isolate DENV-3 | Collection Date | Isolation Source | Genotype | Size (bp) | GenBank Accession Numbers |
| --- | --- | --- | --- | --- | --- | --- |
| 1 | MA37 | 11/14/2019 | Bamako | 3III_B.2 | 1,171 | PX454656 |
| 2 | MA519 | 11/6/2023 | Bamako | 3III_B.2 | 10,649 | PX454657 |
| 3 | MA537 | 11/6/2023 | Bamako | 3III_B.2 | 10,647 | PX454658 |
| 4 | MA626 | 11/15/2023 | Kenieba | 3III_B.2 | 10,649 | PX454659 |
| 5 | MA645 | 11/15/2023 | Koulikoro | 3III_B.2 | 10,648 | PX454578 |
| 6 | MA678 | 11/16/2023 | Bamako | 3III_B.2 | 10,648 | PX454660 |
| 7 | MA680 | 11/16/2023 | Bamako | 3III_B.2 | 10,079 | PX454661 |
| 8 | MA688 | 11/17/2023 | Kenieba | 3III_B.2 | 9,111 | PX454662 |
| 9 | MA742 | 11/21/2023 | Bamako | 3III_B.2 | 10,648 | PX454663 |
| 10 | MA746 | 11/22/2023 | Bamako | 3III_B.2 | 10,648 | PX454664 |
| 11 | MA749 | 11/22/2023 | Kenieba | 3III_B.2 | 10,081 | PX454665 |
| 12 | MA764 | 11/23/2023 | Bamako | 3III_B.2 | 10,648 | PX454666 |
| 13 | MA774 | 11/24/2023 | Kenieba | 3III_B.2 | 10,649 | PX454667 |
| 14 | MA791 | 11/26/2023 | Bamako | 3III_B.2 | 10,649 | PX454668 |
| 15 | MA816 | 11/29/2023 | Bamako | 3III_B.2 | 10,648 | PX454669 |
| 16 | MA828 | 11/29/2023 | Bamako | 3III_B.2 | 10,648 | PX454670 |
| 17 | MA901 | 12/6/2023 | Bamako | 3III_B.2 | 10,649 | PX454671 |
| 18 | MA902 | 12/6/2023 | Bamako | 3III_B.2 | 10,648 | PX454672 |
| 19 | MA934 | 12/8/2023 | Bamako | 3III_B.2 | 10,649 | PX454673 |
| 20 | MA938 | 12/10/2023 | Bamako | 3III_B.2 | 10,648 | PX454674 |
| 21 | MA945 | 12/11/2023 | Bamako | 3III_B.2 | 3,947 | NA |
| 22 | MA975 | 12/14/2023 | Bamako | 3III_B.2 | 10,648 | PX454675 |
| 23 | MA1616 | 10/24/2024 | Bamako | 3III_B.2 | 10,628 | PX454676 |
| 24 | MA1618 | 11/11/2024 | Bamako | 3III_B.2 | 10,649 | PX454677 |
| 25 | MA1619 | 11/20/2024 | Bamako | 3III_B.2 | 10,649 | PX454678 |
| 26 | MA1620 | 11/28/2024 | Bamako | 3III_B.2 | 10,649 | PX454679 |
| 27 | MA1621 | 12/2/2024 | Bamako | 3III_B.2 | 10,649 | PX454680 |

**Table O. GenBank Accession Numbers and Basic Information of the CHIKV Sequences from the Study.**

| Order | ID Isolate CHIKV | Collection Date | Isolation Source | Genotype | Size (bp) | GenBank Accession Numbers |
| --- | --- | --- | --- | --- | --- | --- |
| 1 | MA144 | 09/18/2019 | Mali | West African | 1,642 | PX462883 |
| 2 | MA554 | 11/7/2023 | Bamako | West African | 11,364 | PX453305 |
| 3 | MA1397 | 8/30/2023 | Bamako | West African | 11,823 | PX458876 |
| 4 | MA9999 | 10/30/2023 | Bamako | West African | 11,823 | PX458877 |

**Table P. List of DENV Sequences from French National Reference Center (NRC) for Arboviruses Used in Phylogenetic Analyses.**

| Order | ID Sequence  from NRC | Collection Date | Isolation Source | Genotype | Size (bp) | GenBank Accession Numbers |
| --- | --- | --- | --- | --- | --- | --- |
| 1 | 79650-1-0-1 | 12/14/2024 | Senegal | 1III.A.2 | 10,179 | PX460266 |
| 2 | 78030-1-0-1 | 8/6/2024 | Côte d'Ivoire | 1III.A.2 | 9,245 | PX460267 |
| 3 | 79321-1-0-1 | 11/8/2024 | Senegal | 1III.A.2 | 9,222 | PX460268 |
| 4 | 66232-1-0-1 | 5/21/2023 | Côte d'Ivoire | 1III.A | 8,292 | PX460269 |
| 5 | 66186-1-0-1 | 5/11/2023 | Côte d'Ivoire | 1III.A.2 | 9,058 | PX460270 |
| 6 | 69830-1-0-1 | 9/20/2023 | France | 1III.A | 9,221 | PX460271 |
| 7 | 71213-1-0-1 | 10/24/2023 | France | 1III.A.3 | 9,113 | PX460272 |
| 8 | 75821-1-0-1 | 6/7/2024 | Côte d'Ivoire | 1III.A.2 | 8,178 | PX460273 |
| 9 | 76251-1-0-1 | 6/28/2024 | Togo | 1III.A.2 | 10,179 | PX460274 |
| 10 | 76908-1-0-1 | 8/5/2024 | Mali | 1III.A | 10,178 | PX460275 |
| 11 | 76938-2-0-1 | 8/8/2024 | Cameroon | 1III.A.2 | 10,179 | PX460276 |
| 12 | 26668-1-0-1 | 11/12/2014 | Burkina Faso | 3III.B.2.2 | 10,170 | PX460277 |
| 13 | 25179-1-0-1 | 8/5/2014 | Burkina Faso | 3III.B.2.2 | 10,170 | PX460278 |
| 14 | 70776-1-0-1 | 10/12/2023 | Burkina Faso | 3III.B.2.1 | 10,170 | PX460279 |
| 15 | 71836-1-0-1 | 11/15/2023 | Burkina Faso | 3III.B.2.1 | 10,170 | PX460280 |
| 16 | 51784-1-0-1 | 7/17/2019 | Côte d'Ivoire | 3III.B.2.2 | 10,170 | PX460281 |
| 17 | 51801-1-0-1 | 7/18/2019 | Côte d'Ivoire | 3III.B.2.2 | 10,170 | PX460282 |
| 18 | 58410-1-0-1 | 3/27/2021 | Côte d'Ivoire | 3III.B.2.2 | 10,170 | PX460283 |
| 19 | 59645-1-0-1 | 8/2/2021 | Côte d'Ivoire | 3III.B.2.2 | 9,005 | PX460284 |
| 20 | 60065-1-0-1 | 9/17/2021 | Côte d'Ivoire | 3III.B.2.2 | 10,170 | PX460285 |
| 21 | 62129-1-0-1 | 6/10/2022 | Côte d'Ivoire | 3III.B.2.2 | 9,114 | PX460286 |
| 22 | 24647-1-0-1 | 6/24/2014 | Côte d'Ivoire | 3III.B.2.2 | 10,170 | PX460287 |
| 23 | 44383-1-0-1 | 6/30/2017 | Côte d'Ivoire | 3III.B.2.2 | 10,170 | PX460288 |
| 24 | 25009-1-0-1 | 7/18/2014 | Côte d'Ivoire | 3III.B.2.2 | 10,170 | PX460289 |
| 25 | 72897-1-0-1 | 1/13/2024 | Côte d'Ivoire | 3III.B.2.1 | 10,170 | PX460290 |
| 26 | 76257-1-0-1 | 7/1/2024 | Côte d'Ivoire | 3III.B.2.2 | 10,128 | PX460291 |
| 27 | 17604-1-0-1 | 5/10/2013 | Gabon | 3III.B.2 | 10,170 | PX460292 |
| 28 | 19647-1-0-1 | 10/14/2013 | Togo | 3III.B.2.2 | 10,170 | PX460293 |
| 29 | 43687-1-0-1 | 5/15/2017 | Togo | 3III.B.2.2 | 10,170 | PX460294 |


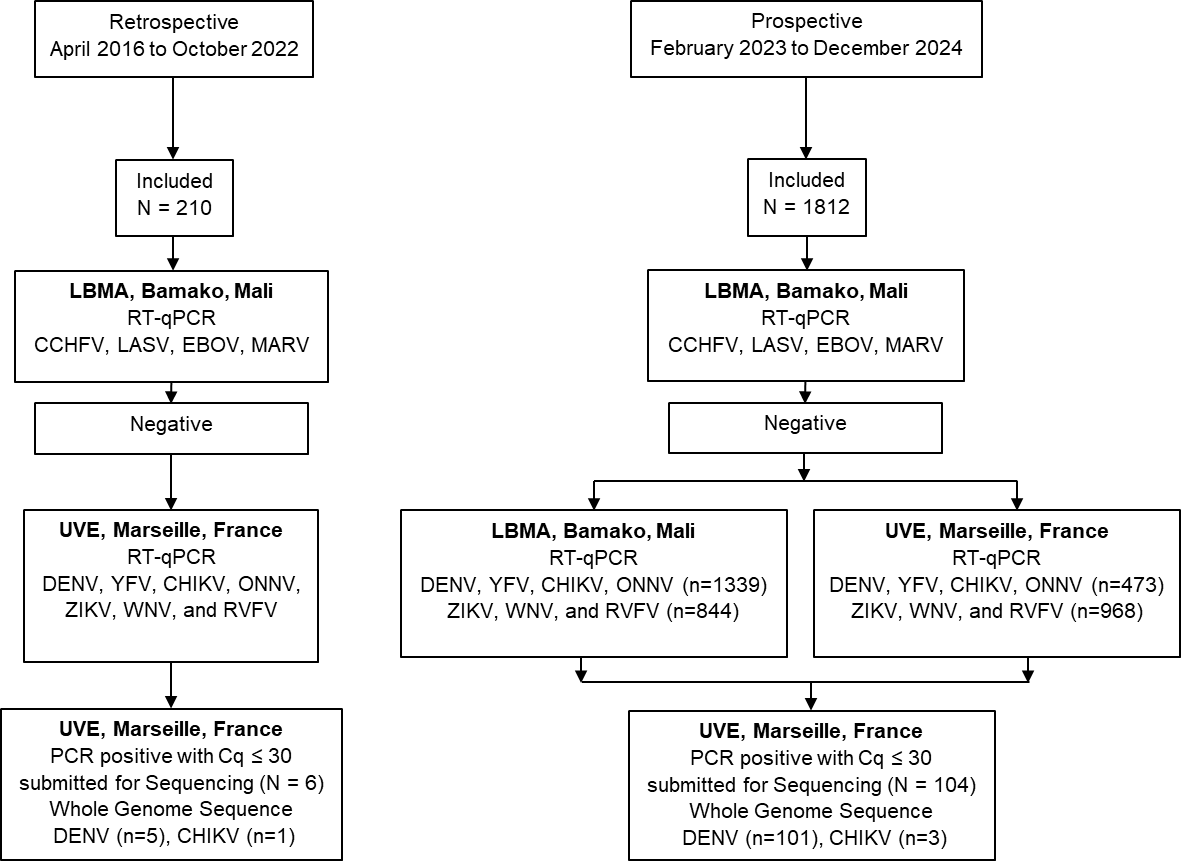


**Fig A. Flowchart of Study Participant’s Enrollment and Sample Processing.**


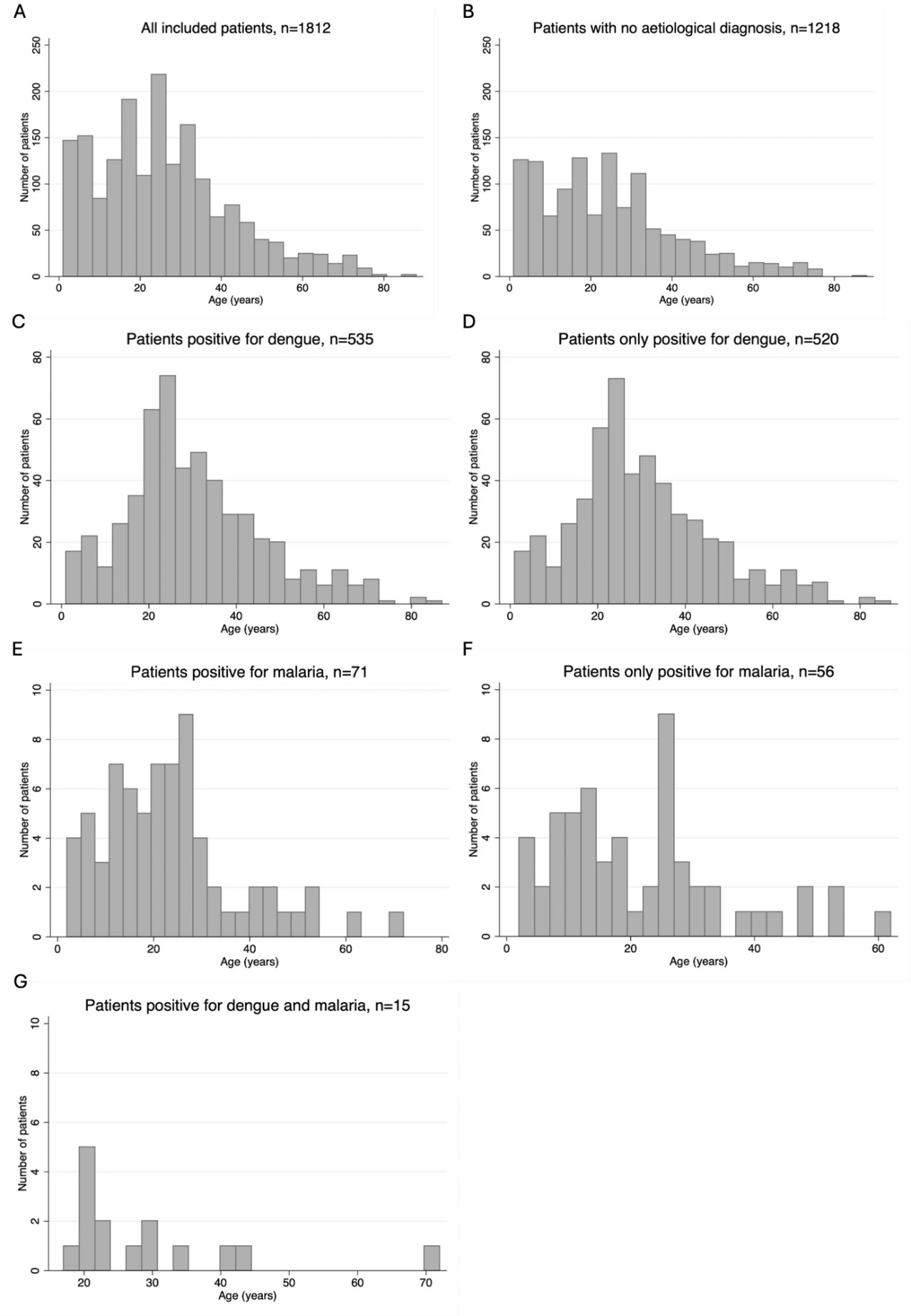


**Fig B. Age Distribution of Patients Enrolled in the Prospective Study.**

A: all included patients. B: Patients with no aetiological diagnosis. C: Patients positives for dengue. D: Patients only positive for dengue. E: Patients positive for malaria. F: Patients only positive for malaria. G: Patients positive for both dengue and malaria.


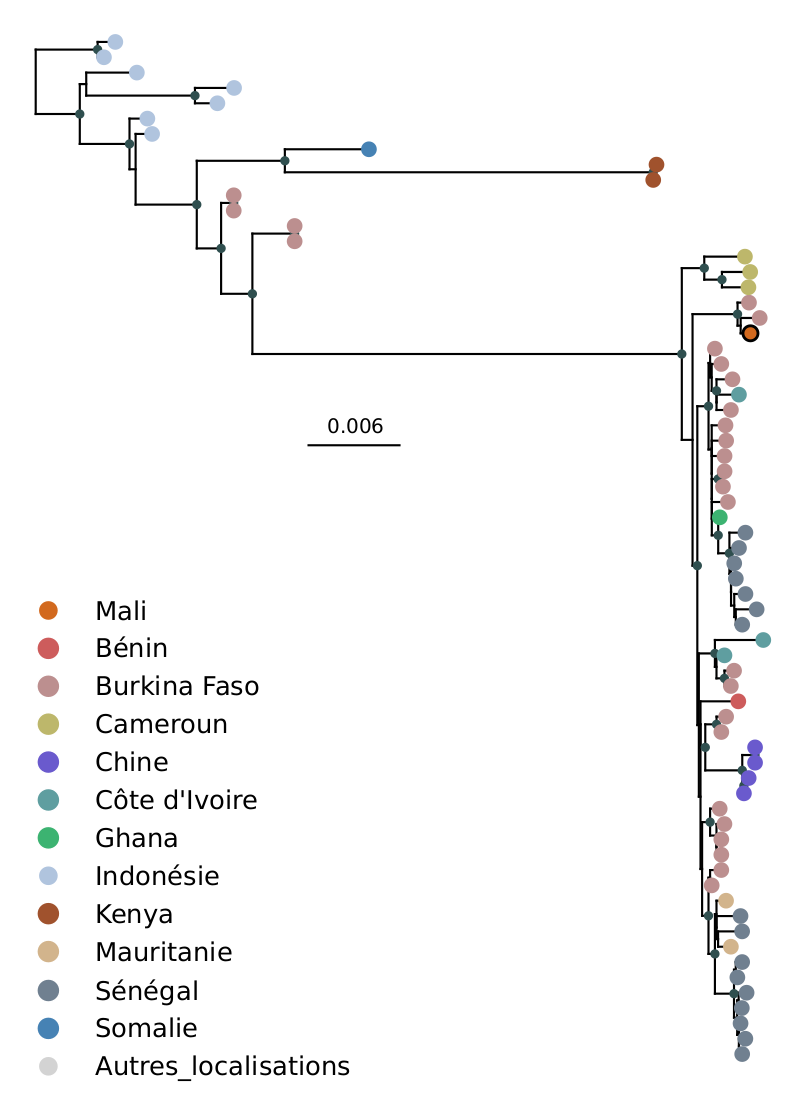


**Fig C. Phylogeny Analysis of DENV-2 Genotype II Clade B Sequences.**

The maximum likelihood (ML) phylogeny was constructed using IQ-Tree (version 1.6.12), with the best-fit model identified by ModelFinder, and branch support estimated using the ultrafast bootstrap approximation (UFBoot2, 1000 replicates). Nodes with bootstrap support above 95 are shown with a black dot. Sequences from this study are shown in orange.


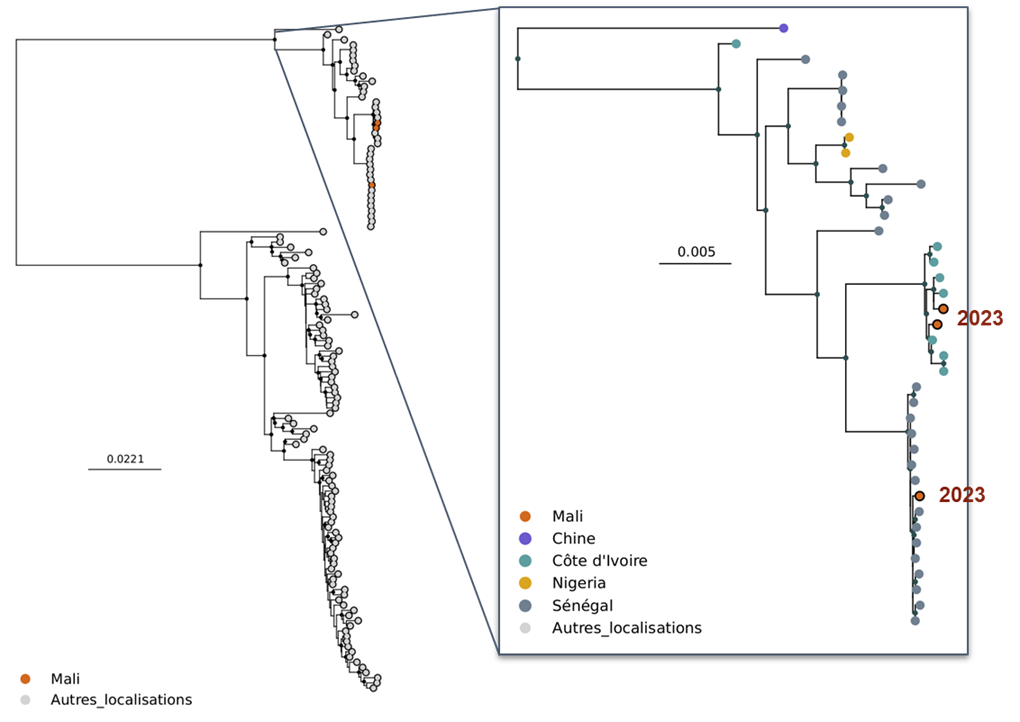


**Figure D. Phylogeny Analysis of CHIKV Sequences.**

The maximum likelihood (ML) phylogeny was constructed using IQ-Tree (version 1.6.12), with the best-fit model identified by ModelFinder, and branch support estimated using the ultrafast bootstrap approximation (UFBoot2, 1000 replicates). Nodes with a bootstrap support above 95 are shown with a black dot. Sequences from this study are shown in orange.


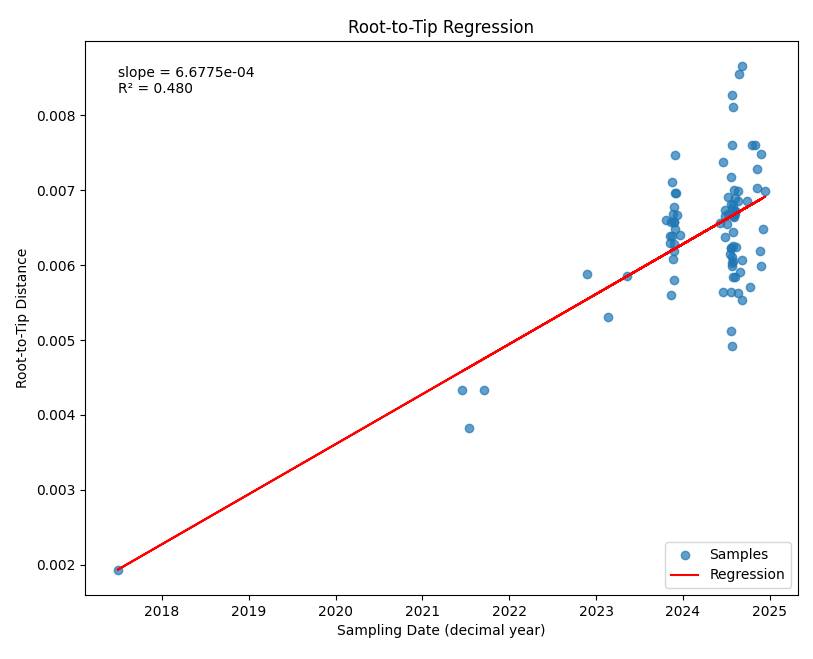


**Fig E. Root-to-tip Analysis of Sequences Used for Bayesian Inference for DENV-1.** Regression of genetic distance against time for all sequences included in the dataset used for bayesian inference for DENV-1.


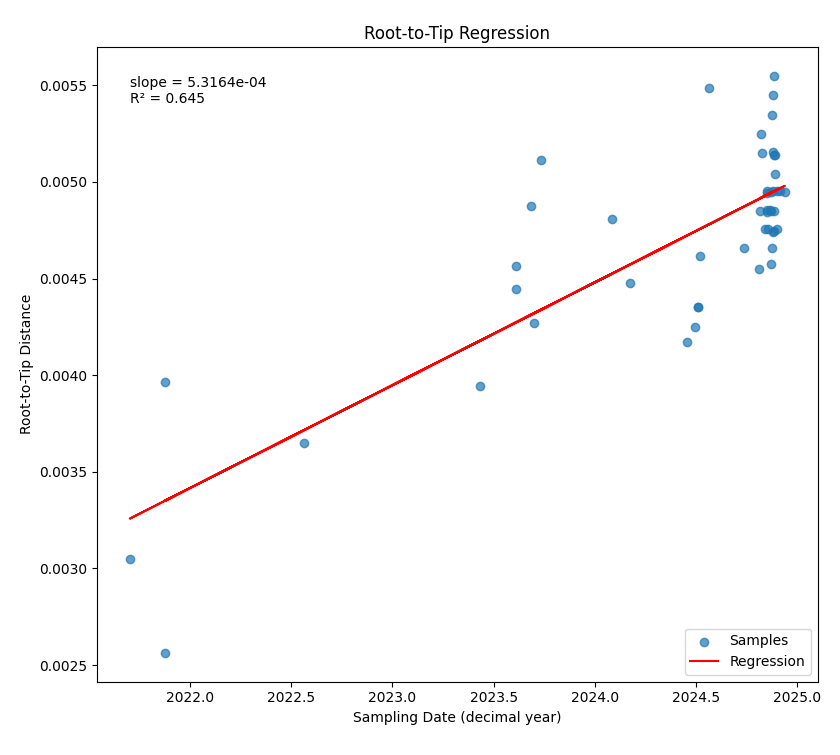


**Fig F. Root-to-tip Analysis of Sequences Used for Bayesian Inference for DENV-2.** Regression of genetic distance against time for all sequences included in the dataset used for bayesian inference for DENV-2.


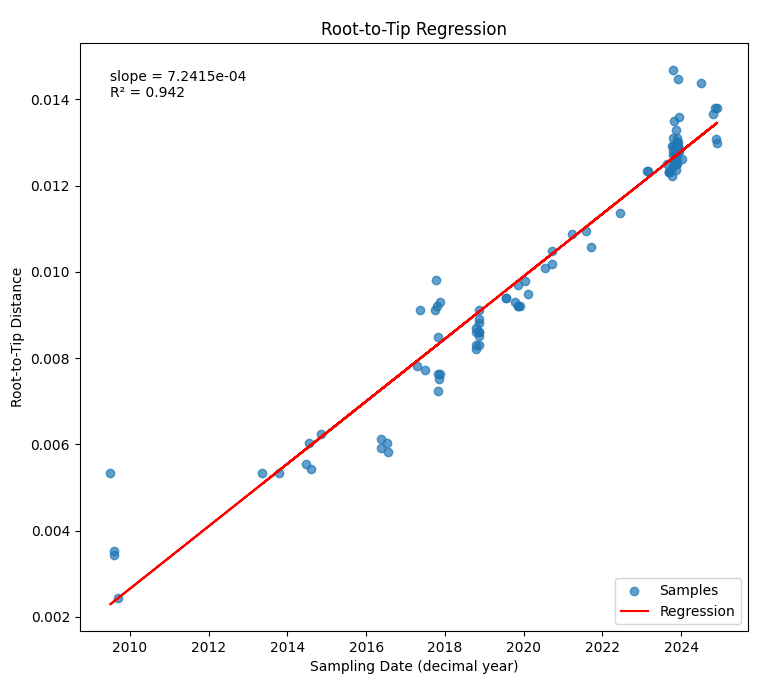


**Fig G. Root-to-tip Analysis of Sequences Used for Bayesian Inference for DENV3.** Regression of genetic distance against time for all sequences included in the dataset used for bayesian inference for DENV-3.

**REFERENCES**

1. Dreier J, Störmer M, Kleesiek K. Use of bacteriophage MS2 as an internal control in viral reverse transcription-PCR assays. J Clin Microbiol. sept 2005;43(9):4551‑7.

2. Liu J, Ochieng C, Wiersma S, Ströher U, Towner JS, Whitmer S, et al. Development of a TaqMan Array Card for Acute-Febrile-Illness Outbreak Investigation and Surveillance of Emerging Pathogens, Including Ebola Virus. J Clin Microbiol. janv 2016;54(1):49‑58.

3. Thirion L, Dubot-Peres A, Pezzi L, Corcostegui I, Touinssi M, de Lamballerie X, et al. Lyophilized Matrix Containing Ready-to-Use Primers and Probe Solution for Standardization of Real-Time PCR and RT-qPCR Diagnostics in Virology. Viruses. 30 janv 2020;12(2):159.

4. Thirion L, Pezzi L, Corcostegui I, Dubot-Pérès A, Falchi A, de Lamballerie X, et al. Development and Evaluation of a Duo Chikungunya Virus Real-Time RT-PCR Assay Targeting Two Regions within the Genome. Viruses. 15 août 2019;11(8):755.

5. Aksamentov I, Roemer C, Hodcroft EB, Neher RA. Nextclade: clade assignment, mutation calling and quality control for viral genomes. J Open Source Softw. 30 nov 2021;6(67):3773.

6. Frumence E, Piorkowski G, Traversier N, Amaral R, Vincent M, Mercier A, et al. Genomic insights into the re-emergence of chikungunya virus on Réunion Island, France, 2024 to 2025. Euro Surveill Bull Eur Sur Mal Transm Eur Commun Dis Bull. juin 2025;30(22):2500344.

7. Ninove L, Nougairede A, Gazin C, Thirion L, Delogu I, Zandotti C, et al. RNA and DNA bacteriophages as molecular diagnosis controls in clinical virology: a comprehensive study of more than 45,000 routine PCR tests. PloS One. 9 févr 2011;6(2):e16142.

8. Wölfel R, Paweska JT, Petersen N, Grobbelaar AA, Leman PA, Hewson R, et al. Virus detection and monitoring of viral load in Crimean-Congo hemorrhagic fever virus patients. Emerg Infect Dis. juill 2007;13(7):1097‑100.

9. Nikisins S, Rieger T, Patel P, Müller R, Günther S, Niedrig M. International external quality assessment study for molecular detection of Lassa virus. PLoS Negl Trop Dis. mai 2015;9(5):e0003793.

10. Gibb TR, Norwood DA, Woollen N, Henchal EA. Development and evaluation of a fluorogenic 5’ nuclease assay to detect and differentiate between Ebola virus subtypes Zaire and Sudan. J Clin Microbiol. nov 2001;39(11):4125‑30.

11. Huang Y, Wei H, Wang Y, Shi Z, Raoul H, Yuan Z. Rapid detection of filoviruses by real-time TaqMan polymerase chain reaction assays. Virol Sin. oct 2012;27(5):273‑7.

12. Weidmann M, Mühlberger E, Hufert FT. Rapid detection protocol for filoviruses. J Clin Virol Off Publ Pan Am Soc Clin Virol. mai 2004;30(1):94‑9.

13. Panning M, Grywna K, van Esbroeck M, Emmerich P, Drosten C. Chikungunya fever in travelers returning to Europe from the Indian Ocean region, 2006. Emerg Infect Dis. mars 2008;14(3):416‑22.

14. Wesselmann KM, Luciani L, Thirion L, de Lamballerie X, Charrel R, Pezzi L. Analytical and clinical evaluation of a duplex RT-qPCR assay for the detection and identification of o’nyong-nyong and chikungunya virus. Emerg Microbes Infect. déc 2024;13(1):2429650.

15. Huhtamo E, Hasu E, Uzcátegui NY, Erra E, Nikkari S, Kantele A, et al. Early diagnosis of dengue in travelers: comparison of a novel real-time RT-PCR, NS1 antigen detection and serology. J Clin Virol Off Publ Pan Am Soc Clin Virol. janv 2010;47(1):49‑53.

16. Leparc-Goffart I, Baragatti M, Temmam S, Tuiskunen A, Moureau G, Charrel R, et al. Development and validation of real-time one-step reverse transcription-PCR for the detection and typing of dengue viruses. J Clin Virol Off Publ Pan Am Soc Clin Virol. mai 2009;45(1):61‑6.

17. Domingo C, Patel P, Yillah J, Weidmann M, Méndez JA, Nakouné ER, et al. Advanced yellow fever virus genome detection in point-of-care facilities and reference laboratories. J Clin Microbiol. déc 2012;50(12):4054‑60.

18. Corman VM, Rasche A, Baronti C, Aldabbagh S, Cadar D, Reusken CB, et al. Assay optimization for molecular detection of Zika virus. Bull World Health Organ. 1 déc 2016;94(12):880‑92.

19. Linke S, Ellerbrok H, Niedrig M, Nitsche A, Pauli G. Detection of West Nile virus lineages 1 and 2 by real-time PCR. J Virol Methods. déc 2007;146(1‑2):355‑8.

20. Tang Y, Anne Hapip C, Liu B, Fang CT. Highly sensitive TaqMan RT-PCR assay for detection and quantification of both lineages of West Nile virus RNA. J Clin Virol Off Publ Pan Am Soc Clin Virol. juill 2006;36(3):177‑82.

21. Drosten C, Göttig S, Schilling S, Asper M, Panning M, Schmitz H, et al. Rapid detection and quantification of RNA of Ebola and Marburg viruses, Lassa virus, Crimean-Congo hemorrhagic fever virus, Rift Valley fever virus, dengue virus, and yellow fever virus by real-time reverse transcription-PCR. J Clin Microbiol. juill 2002;40(7):2323‑30.

22. Weidmann M, Sanchez-Seco MP, Sall AA, Ly PO, Thiongane Y, Lô MM, et al. Rapid detection of important human pathogenic Phleboviruses. J Clin Virol Off Publ Pan Am Soc Clin Virol. févr 2008;41(2):138‑42.

23. Baronti C, Piorkowski G, Leparc-Goffart I, de Lamballerie X, Dubot-Pérès A. Rapid next-generation sequencing of dengue, EV-A71 and RSV-A viruses. J Virol Methods. 15 déc 2015;226:7‑14.
